# *Naegleria* genus pangenome reveals new structural and functional insights into the versatility of these free-living amoebae

**DOI:** 10.3389/fmicb.2022.1056418

**Published:** 2023-02-01

**Authors:** Alexis Dereeper, Nina Allouch, Vincent Guerlais, Maëlle Garnier, Laurence Ma, Johan F. De Jonckheere, Sandeep J. Joseph, Ibne Karim M. Ali, Antoine Talarmin, Isabel Marcelino

**Affiliations:** ^1^Institut Pasteur de la Guadeloupe, Unité TReD-Path, Les Abymes, Guadeloupe, France; ^2^Institut Pasteur de Paris, Biomics, Paris, France; ^3^Scientific Institute of Public Health, Brussels, Belgium; ^4^Centers for Disease Control and Prevention (CDC), Atlanta, GA, United States

**Keywords:** free-living amoebae, *Naegleria*, whole genome sequencing, genome plasticity, pangenome, core genome, species-specific genes, *Naegleria fowleri* virulence-associated genes

## Abstract

**Introduction:**

Free-living amoebae of the *Naegleria* genus belong to the major protist clade Heterolobosea and are ubiquitously distributed in soil and freshwater habitats. Of the 47 *Naegleria* species described, *N. fowleri* is the only one being pathogenic to humans, causing a rare but fulminant primary amoebic meningoencephalitis. Some *Naegleria* genome sequences are publicly available, but the genetic basis for *Naegleria* diversity and ability to thrive in diverse environments (including human brain) remains unclear.

**Methods:**

Herein, we constructed a high-quality *Naegleria* genus pangenome to obtain a comprehensive catalog of genes encoded by these amoebae. For this, we first sequenced, assembled, and annotated six new *Naegleria* genomes.

**Results and Discussion:**

Genome architecture analyses revealed that *Naegleria* may use genome plasticity features such as ploidy/aneuploidy to modulate their behavior in different environments. When comparing 14 near-to-complete genome sequences, our results estimated the theoretical *Naegleria* pangenome as a closed genome, with 13,943 genes, including 3,563 core and 10,380 accessory genes. The functional annotations revealed that a large fraction of *Naegleria* genes show significant sequence similarity with those already described in other kingdoms, namely Animalia and Plantae. Comparative analyses highlighted a remarkable genomic heterogeneity, even for closely related strains and demonstrate that *Naegleria* harbors extensive genome variability, reflected in different metabolic repertoires. If *Naegleria* core genome was enriched in conserved genes essential for metabolic, regulatory and survival processes, the accessory genome revealed the presence of genes involved in stress response, macromolecule modifications, cell signaling and immune response. Commonly reported *N. fowleri* virulence-associated genes were present in both core and accessory genomes, suggesting that *N. fowleri*’s ability to infect human brain could be related to its unique species-specific genes (mostly of unknown function) and/or to differential gene expression. The construction of *Naegleria* first pangenome allowed us to move away from a single reference genome (that does not necessarily represent each species as a whole) and to identify essential and dispensable genes in *Naegleria* evolution, diversity and biology, paving the way for further genomic and post-genomic studies.

## 1. Introduction

Members of the *Naegleria* genus belong to the major eukaryotic lineage Heterolobosea, that deviated from other eukaryotic lineages over a billion years ago (Fritz-Laylin et al., 2010). They are ubiquitous in soils and freshwater habitats and are important predators of cyano-and eubacteria, hereby regulating bacterial populations in lakes and rivers (De Jonckheere, 2011). Some species can naturally grow at 37°C, and others can grow up to 45°C (De Jonckheere, 2014). *Naegleria* usually have two developmental stages: the trophozoite (which is the metabolically active form in which they can move, feed and multiply) and the cysts (the dormant and resistant form); some species can transform into flagellates, allowing the amoeba to rapidly move around and look for more favorable conditions (Fritz-Laylin et al., 2010; De Jonckheere, 2014). The *Naegleria* genus currently contains 47 recognized species (De Jonckheere, 2011) but only *N. fowleri* (also popularly known as “brain-eating amoeba”) is a confirmed human pathogen, causing primary amoebic meningoencephalitis (PAM).

PAM is a rare but fatal disease (with a 95% mortality rate), affecting mainly healthy children or young adults (Sarink et al., 2022). Infection occurs when contaminated water enters the nose, *N. fowleri* (specially trophozoites) follows the olfactory nerve to the brain through the cribriform plate. There, it induces phagocytosis of brain material, provoking tissue damage and hemorrhagic necrosis causing a fatal brain infection. The disease progresses rapidly leading to death within 7–12 days (Moseman, 2020). Combined with its low incidence (Trabelsi et al., 2012; Siddiqui et al., 2016), early diagnosis is difficult as the PAM symptoms closely resembled bacterial meningitis (Jahangeer et al., 2020); the link with *Naegleria* is usually made post-mortem by microscopic examination of the cerebral spinal fluid or by conventional or real-time PCR. In recent years, an increased number of PAM cases have been reported worldwide, in particular in temperate regions and developing countries; this is probably due to global warming, global overpopulation and increased industrial activities (Kemble et al., 2012; Siddiqui et al., 2016; Maciver et al., 2020). In the Caribbean region, the first fatal case of *N. fowleri* was reported in a geothermal bath in Guadeloupe in 2008 (Nicolas et al., 2010). Despite successful treatment in a very few cases with miltefosine and other antimicrobial medication (Debnath, 2021), the same antibiotic regime failed in other cases, suggesting the need to find effective therapies (Khan et al., 2021). Several studies have shown that *N. fowleri* pathogenesis involves both contact-dependent interaction with the host (through brain damaging, sucker-like surface structure called “food cup” which enables *N. fowleri* to interact with the host extracellular matrix (ECM) through a process of adhesion, invasion and degradation of ECM and nerve cell) and contact independent interaction (through the release of different proteases with proteolytic function and hydrolysing activity, that in central nervous system, cause further destruction of nerves; Jamerson et al., 2012; Herman et al., 2021; Rodriguez-Anaya et al., 2021; Sarink et al., 2022). Despite such work, the pathogenic factors of *N. fowleri* are still unclear.

At the moment, over 60 *Naegleria* genome sequences are publicly available (with different levels of completeness and using different sequencing methodologies) for the non-pathogenic species *N. gruberi* (Fritz-Laylin et al., 2010) and *N. lovaniensis* (Liechti et al., 2018; Joseph et al., 2021), and for the pathogenic *N. fowleri* (Zysset-Burri et al., 2014; Ali et al., 2021; Herman et al., 2021; Joseph et al., 2021). Comparative genomic studies were already performed within *N. fowleri* species (Joseph et al., 2021) and between *Naegleria* species (Liechti et al., 2018; Herman et al., 2021) but they do not describe the complete gene landscape of a *Naegleria* species or genus because of the large numbers of variations between accessions.

To better understand *Naegleria* genome evolution, phenotypic diversity and versatility, we aimed to construct the first high-quality genus pangenome for *Naegleria* that would list the core genes (involved in housekeeping and conserved survival processes) and dispensable genes that are present only in a subset of species (being responsible for phenotypic differences between isolates and may be involved in pathogenesis). For this, we first sequenced and annotated three new *N. fowleri* strains (NF_AR12 from United States, NF_PA34 from Australia and NF_NF1 from Guadeloupe, with different genotypes and all being environmental isolates) and three new *N. lovaniensis* strains: NL_F9 (from Belgium), NL_Lova6 and NL_Lova7 (both isolated in Guadeloupe). Afterwards, we compared these 6 new genomes to available genomic data with different levels of completeness, from different *Naegleria* species (and strains) isolated from distinct geographical regions and environments (water, soil, human).

## 2. Materials and methods

### 2.1. Amoebae samples

The biological samples used for Illumina sequencing are presented in Table 1. DNA samples originated from *N. fowleri* AR12 and PA34, and *N. lovaniensis* F9 were kindly provided by Professor J. F. De Jonckheere and produced as described elsewhere (De Jonckheere, 1998); the others 3 strains (1 *N. fowleri* and 2 *N. lovaniensis*) were obtained as described in the following section. For pangenome and comparative genomics analyses, we also included 8 assembled and annotated whole-genome sequences of *Naegleria* genus published (Table 1). At the time of writing this paper, 49 draft genome sequences of *N. fowleri* species (from clinical and environmental origin; Joseph et al., 2021) were also available in the NCBI’s Sequence Read Archive (SRA). These sequences (Supplementary Table S1) were downloaded and used for whole-genome SNPs phylogenetic analysis (as described in Section 2.7.1).

**Table 1:** Genome assemble and annotation statistics of published and newly sequenced genomes of *Naegleria* sp.

*Species*	*N. gruberi*	*N. fowleri*								*N. lovaniensis*				
Strain	NEG-M ATCC 30224	NF_ATCC 30894 (Lee)	NF_ATCC 30863 (Carter)	NF_V212	NF_986	NF_TY* (ATCC 30107)	NF_NF1	NF_AR12	NF_PA34	NL_ATCC 30569	NL_76–15-250	NL_F9	NL_Lova6	NL_Lova7
Genotype	–	3	2	2	5	3	3	2	5	n/a	n/a	n/a	n/a	n/a
Origin	Environ. (soil)	Clinical			Environ. (water)	Clinical	Environmental (water)			Environmental (water)				
Geographical location	United States	United States			Australia	United States	Guadeloupe	United States	Australia	United States	Belgium	Belgium	Guadeloupe	
Year of origin	1969	1968	1978	1990	–	1969	2018	1976	1972	1970	1976	1980	2018	2018
Sequencing technology	Sanger	Nanopore	Illumina/Roche 454	Illumina/Roche 454	Illumina	Illumina/PacBio	Illumina	Illumina	Illumina	PacBio	PacBio	Illumina	Illumina	Illumina
Genome size (Mbp)	40.9	29.5	29.6	27.7	27.5	27.9	27.6	27.3	27.3	30.8	30.8	27.7	26.9	26.5
GC content (%)	35	36.9	35	36	36	36.9	36.9	36.9	36.9	37	36.3	36.9	37	37
Number of scaffolds (anchored in N chromosomes)	1,977	90	2,530	1962	1919	37	500 (37)	656 (37)	534 (37)	111	199	754 (37)	1,959 (37)	2,384 (37)
N50 of scaffolds (bp)	159,679	717,491	38,800	86,051	45,674	756,811	125,650	81,421	112,144	657,933	455,122	78,479	26,705	17,708
Number of predicted genes (ORFs)	16,620	13,925	11,499	12,677	11,599	9,405	9,336	9,441	11,036	15,195	11,305	9,481	9,578	9,305
Average gene length (bp)	1,677	–	1984	1785	1955	–	3,003	2,946	2,284	–	–	3,283	3,010	3,034
Coding (%)	57.8	–	70.79	71.35	73.01	68	59.4	58.5	68.5	–	65	57.5	60	61.5
Repeat content (%)	5.1	6	2.5	–	–	5.3	5	3.3	1.4	3.5	10.8	2.7	2.4	2.4
Complete BUSCOs (%)	85.7	86.5	87.8	88.3	87.9	84.3	82.0	84.8	87.2	85.5	78.8	88.3	87.2	86.0
Fragmented BUSCOs	1.3	2.3	2.7	2.4	3.1	2.8	6.7	4.1	2.9	2.6	5.1	4.7	3.5	4.1
BUSCO Missing	13	11.2	9.5	9.3	9.0	12.9	11.3	11.1	9.9	11.9	16.1	7.0	9.3	9.9
Reference	Herman et al. (2021)	Liechti et al. (2019)	Herman et al. (2021)			Ali et al. (2021) and Joseph et al. (2021)	This work			Liechti et al. (2018)	Joseph et al. (2021)	This work		

“–” stands for not-available while “n/a” stands for non-applicable.

* Out of 52 *N. fowleri* genomes sequenced and presented by Joseph et al. (2021), we only shown only for the “close-to-complete” genome of the *N. fowleri* TY isolate.

#### 2.1.1. Amoebae isolation and identification (Guadeloupean strains)

Water samples (1 L) were collected from 3 geothermal baths in Guadeloupe: Curé (Bouillante), Dolé (Gourbeyre) and La Lise (Bouillante) and treated as previously described (Moussa et al., 2013, 2020). Amoebae cultivated in NNA-*E.coli* were recovered by scrapping the agar plate with 800 μL of T1 Lysis Buffer (from the kit NucleoSpin Tissue, Macherey Nagel, Germany). Afterwards, the samples went through a DNA extraction protocol (NucleoSpin^®^ Tissue DNA extraction kit, Macherey-Nagel), following the manufacturer’s recommendations. DNA was stored at −20°C until use. Amoebae identification was performed by PCR using ITS primers, as described elsewhere (Moussa et al., 2013). ITS amplicons Sanger sequencing was performed at Eurofins Genomics (Germany). A homology search was performed with BLAST software from the National Center for Biotechnology Information homepage.^1^ The sequence data obtained were aligned by ClustalW software^2^ with the sequences of *Naegleria* species are presented in Supplementary Table S2 and were also deposited in GenBank.^3^

For whole genome sequencing purposes, NF_NF1 (5 biological replicates), NL_Lova6 and NL_Lova7 were cultured in axenic culture during at least 5 passages in tissue culture flasks using the SCGYEM conditions (De Jonckheere, 1977). Trophozoites (1 × 10^6^ *Naegleria* per strain) cultivated in axenic culture conditions were scraped from T-flasks and centrifuged at 1,000*g* for 10 min at room temperature; after supernatant removal, 200 μL of T1 buffer was added. “T1-cell suspension” was kept at −20°C until further use. For genome Illumina sequencing, we included a RNAse step during the extraction protocol, following the manufacturer’s recommendations.

### 2.2. Whole genome sequencing

DNA samples were used for library preparation using TruSeq DNA PCR-free library prep kit (following fragmentation into 350 bp-long fragments) and TruSeq DNA UD Indexes (Illumina). Paired-end sequencing was performed on a MiSeq system with a nano v2 flowcell and reagents for 2 × 151 cycles (Illumina). Whole genome Illumina sequencing was performed at the Biomics Core Facility (Institut Pasteur, Paris, France). Number of reads by experiment or replicate are presented in Supplementary Table S3.

### 2.3. Bioinformatics analyses

#### 2.3.1. Whole-genome SNP-based phylogenetic analysis

A whole-genome phylogenetic analysis based on SNPs was performed for *N. fowleri* and *N. lovaniensis*. For the comparison of *N. fowleri* strains, we compiled our 11 samples (including the 5 biological replicates of NF_NF1) with the published raw data publicly available from SRA (*N* = 49; Supplementary Table S1; Joseph et al., 2021). For *N. lovaniensis*, we used our 3 samples plus the reads from the sequencing projects of the strains NL_ATCC 30569 (Liechti et al., 2018) and NL_76-15-250 (Joseph et al., 2021). After a cleaning of raw reads using Cutadapt, Illumina raw reads were first mapped either against the *N. fowleri* TY “close-to-complete” genome (Ali et al., 2021) or the *N. lovaniensis* ATCC 30569, using BWA-MEM (version 0.7.17-r1188) software (Li, 2013). BAM mapping files were then converted to pileup format using SAMtools (Danecek et al., 2021) and SNP calling was performed using VarScan (Koboldt et al., 2012) for each sample, using a minimum read coverage of 8X with a Phred quality score of at least 15. Genetic variations and alleles were then compiled into a global SNP matrix file (VCF file), using a home-made script. SNP-based phylogenetic tree was then generated using the SNiPlay web application (Dereeper et al., 2015). Finally, the phylogenetic tree was displayed using the iTOL v6 online application (Letunic and Bork, 2021). In addition, *N. fowleri* samples were assigned to internal transcribed space (ITS) genotype, using a home-made script searching for ITS genotyping loci directly from raw reads.

#### 2.3.2. Genome assembly

In order to estimate the conservation of scaffolds between genomes from different species, we first aligned the published scaffolds of *N. lovaniensis* (ATCC 30569) (Liechti et al., 2018) against the recently published genome of *N. fowleri* TY (Ali et al., 2021), which consists of 37 well defined pseudo-chromosomes. For this, we used the D-genies online application (Cabanettes and Klopp, 2018), taking advantage of minimap2 alignment software (Li, 2018). Except for minor rearrangements detected in only one pseudo-chromosome, a good collinearity was observed between *N. lovaniensis* scaffolds and *N. fowleri* pseudo-chromosomes (Supplementary Figure S1), suggesting that scaffolds from the 2 species can be simply ordered according to their matching positions in pseudo-chromosomes. This data manipulation prevents the generation of heterogeneous outputs for the different genomes and will facilitate thereafter the comparison of genomes and their graphical representation, notably for synteny analysis. The 6 new *Naegleria* genomes were assembled using the same strategy. Briefly, after a cleaning step by cutadapt, filtered reads were de-novo assembled using SPAdes (Prjibelski et al., 2020), to generate scaffolds independently of the respective reference genome and ensure subsequent microsynteny analysis. In a second step, scaffolds were anchored, ordered and oriented, using the 37 chromosomes of *N. fowleri* TY by running the RaGOO program (Alonge et al., 2019).

#### 2.3.3. Ploidy and heterozygosity analyses based on SNPs

An estimate of ploidy level was performed for each of the newly sequenced genomes, based on the ratio of alleles of SNPs. For this purpose, cleaned reads were mapped back against the corresponding assembly of the genome with BWA mem, and SNPs were called with the same approach as previously with the combination SAMtools mpileup/VarScan. A home-made script was developed for evaluating the level of heterozygosity, and for extracting the information of allele depth ratio (i.e., between reference and alternate allele) at each heterozygous position. Allele ratios were then reported along the chromosomes using Circos visualization (Krzywinski et al., 2009) and the distribution of ratios was calculated for each genome. This technique allows to evaluate the global level of ploidy as well as its variation along the chromosomes and thus to detect potential abnormality in specific chromosomes or genomic regions.

#### 2.3.4. Gene prediction and annotation

A complete Galaxy annotation workflow based on iterative runs of MAKER2 annotation pipeline (Holt and Yandell, 2011) was constructed and implemented on Galaxy KaruBioNet (Couvin et al., 2022) and applied for the annotation of each new genome, independently. The protein-coding gene annotation by MAKER2 combines homology prediction, *ab initio* prediction [using SNAP (Korf, 2004) and AUGUSTUS (Hoff and Stanke, 2013)], and full-length transcriptome prediction based on NGS sequencing. Transcriptomic resources were prepared separately for *N. fowleri* and *N. lovaniensis* genomes, from public RNASeq datasets downloaded from European Nucleotide Archive (ENA), PRJNA642022 project, using, respectively, samples NF_Nelson_medium with NF_PYNH_medium (3 replicates each) and NL_Nelson_medium with NL_PYNH_medium (3 replicates each; Zysset-Burri et al., 2014; Supplementary Table S1). For each, RNASeq raw reads were cleaned using Cutadapt (Martin, 2011), mapped against the genome using MapSplice (Wang et al., 2010) and a genome-guided de-novo transcriptome assembly was constructed by Trinity (trinityrnaseq; Grabherr et al., 2011) using the –*genome_guided_bam* option. Assemblies were then gathered and concatenated by species to be subsequently provided as EST evidence for gene prediction; herein, we used 183,318 and 97,050 ESTs for *N. fowleri* and *N. lovaniensis*, respectively. In this work, 3 rounds of MAKER2 were performed. At each round, the gene annotation obtained was evaluated by trained gene predictors SNAP and AUGUSTUS. The new gene models were re-used in the next round of MAKER2 to improve the annotation and create a weighted consensus of the gene structures. Repeat sequences were annotated by both Repbase and a custom repeat library. The Repbase library (Jurka, 2000) was downloaded from https://www.girinst.org/server/RepBase/, and the custom repeat library was constructed on each genome sequence by using RepeatModeler (version 2.0.1; Flynn et al., 2020). These two libraries were concatenated and provided to RepeatMasker (Tarailo-Graovac and Chen, 2009) as implemented in MAKER2, to identify repetitive elements. Subsequently, the annotation completeness was evaluated with BUSCO (Simão et al., 2015). Finally, the density of genes, exons and repeats were calculated from GFF annotation files thanks to a home-made script.

#### 2.3.5. Functional annotation

Gene functions were assigned according to the best alignment of predicted protein sequences using BlastP (default values: E-value 1E-03) to the Uniprot database (including the SWISS-PROT and TrEMBL databases). We used InterProScan program (Jones et al., 2014) to assign conserved protein motifs (PFAM, InterPro). An additional assignation of predicted proteins to specific COG (Clusters of Orthologous Groups) was performed independently using the “COG assignation” Galaxy wrapper available in Galaxy KaruBioNet,^4^ (Couvin et al., 2022), based on rpsblast and cdd2cog Perl script.

#### 2.3.6. Pangenome analysis

To perform the pangenome analysis of *Naegleria* genus, we used a dataset of 14 *Naegleria* genomes with clinical and environmental isolates from distinct geographic location, 8 genomes of *N. fowleri* (NF_ATCC 30894, NF_ATCC 30863, NF_V212, NF_986, NF_TY, NF_NF1, NF_AR12 and NF_PA34), 5 genomes of *N. lovaniensis* (NL_ATCC 30569, NL_76–15-250, NL_F9, NL_Lova6 and NL_Lova7) and one of *N. gruberi* (strain NEG-M-ATCC 30224; Table 1).

For this, published protein FASTA files containing predicted protein sequences were downloaded either from the AmoebaDB resource^5^ (release 53) or from the NCBI FTP server, and were compiled together with the MAKER2 output files generated for our 6 new genomes to perform a genomic and protein comparison.

The pangenome analysis was conducted using OrthoFinder software (Emms and Kelly, 2019) with all-versus-all BLAST strategy to define the orthogroups among the 14 genomes. The resulting presence/absence matrix was analyzed with an in-house developed Perl script to extract and classify genes as core-genes (genes present in all *Naegleria* sp.) and accessory genes (genes present at least once in 1 or more but not all *Naegleria* species). In this latter category, we emphasize on species-specific genes which can be subdivided in 2 gene sets: species-core specific genes (gene shared by all strains within one *Naegleria* species) and species-specific accessory genes (genes present at least once in 1 or more strains of one *Naegleria* species). To determine whether the pangenome can be considered closed or open, we calculated the alpha parameter using the MicroPan R library (Snipen and Liland, 2015).

#### 2.3.7. Synteny analysis on *Naegleria* core genome

Chromosomal synteny was estimated by connecting core-genes between two representative genomes, NF_NF1 and NL_Lova7 for *N. fowleri* and *N. lovaniensis,* respectively. A home-made Perl script was used to extract core-gene locations and identify (i) specific links that connect core-genes located in different chromosomes between the two species, and (ii) links that connect core-genes that physically distant more than 50 kb between homologous chromosomes to highlight insertion or inversion. Syntenic regions shared between pairs of homologous chromosomes were visualized using both Circos (Krzywinski et al., 2009) and Mauve Viewer.^6^

#### 2.3.8. Protein–protein interaction network

Schematic information on *Naegleria* biological pathways is mainly available for *N. gruberi*.^7^ However, based on our pangenome results, several genes present in NG are absent in NF and NL (and *vice-versa*) and many *Naegleria* genes are of unknown function. Herein, in attempt to bring new insights on *Naegleria* biological pathways, we used Cytoscape v3.8.0 software platform (Su et al., 2014) combined with StringApp (Doncheva et al., 2019) plugin. STRING is a database of quality-controlled protein–protein association networks and enables researchers to construct a functional association network of uploaded genes/proteins of an organism based on three aspects: computational prediction, from knowledge transfer between organisms, and from interactions aggregated from other (primary) databases (Szklarczyk et al., 2019). From the 13,972 genes in *Naegleria* pangenome (Supplementary Table S4), we first constructed a network consisting of 8,829 protein nodes and 44,347 edges (data not shown), and then, we selected only the connected nodes to create a Protein–Protein Interaction (PPI) Network (PPIN) with 3,970 nodes and 44,314 edges. The enrichment analysis using StringApp was performed with a high confidence score of 0.75 and based on non-redundant terms (threshold above 0.75) of Gene Ontology (GO) term, Kyoto encyclopedia of genes and genomes (KEGG) pathway data and Reactome Pathways Functional Interaction (FI) Network. This allowed us to group genes/proteins according to their biological function.

## 3. Results and discussion

### 3.1. *Naegleria* phylogenic structure

**Figure 1:**
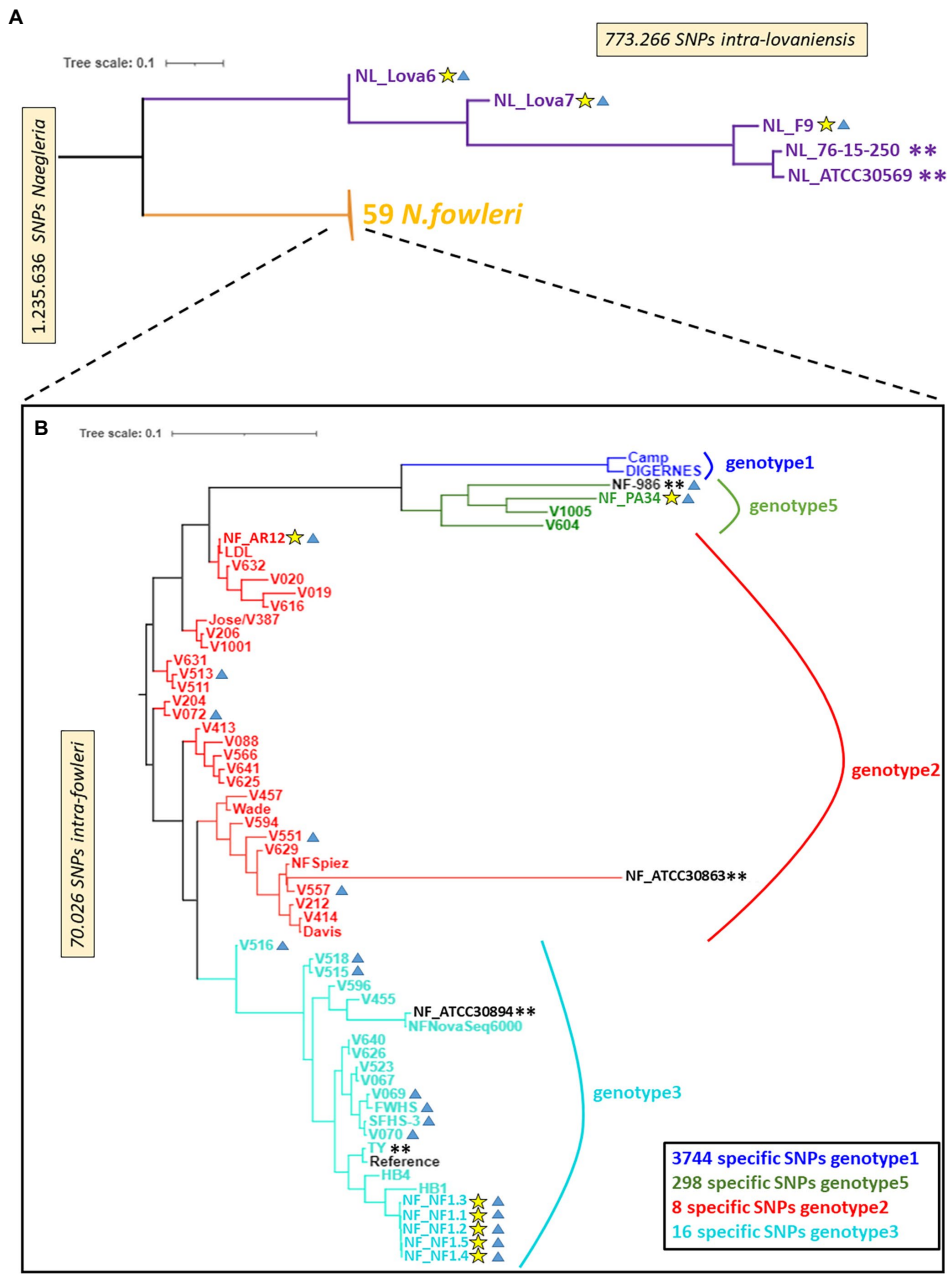
A neighbor-joining phylogenomic tree of *Naegleria fowleri* and *Naegleria lovaniensis* strains using the whole-genome SNPs. Phylogenomic trees are, respectively, based on 1,235,636 SNPs found in *Naegleria* genus **(A)** and 70,026 SNPs found in *N. fowleri* species **(B)**. Yellow stars indicate strains sequenced in the present study, blue triangles indicate environmental strains, and double asterisks indicate strains for which a reference genome assembly and annotation were available at the date of writing. Bootstrap support estimates of major ancestral nodes are also shown.

A description of the phylogenetic relationships within the genus *Naegleria* using different methods has been previously reported (Pelandakis et al., 2000; De Jonckheere, 2002; Joseph et al., 2021). Herein, we sequenced, assembled, and annotated new genomes from *N. fowleri* and *N. lovaniensis* species, and searched for genome-wide SNPs between strains of these two species (Figure 1; Supplementary Table S1). This measure of phylogenetic diversity has proven to be useful to discriminate among closely related organisms and help resolve both short and long branches in a tree (as reviewed by Ngoot-Chin et al., 2021). The reads associated with each sample were mapped to the *N. fowleri* TY reference genome (Ali et al., 2021) and a SNP calling process was performed. A total of 1,200,000 high-quality reference-based SNPs were detected across the 65 *Naegleria* genomes analyzed. Our results show that (i) additional clades were created when we included our new *N. lovaniensis* strains and (ii) *N. lovaniensis* is more diverse than *N. fowleri*, as 773,266 SNPs were detected within intra-*N. lovaniensis* species against only 70,026 SNPs within intra-*N. fowleri*.

By restricting the SNP matrix and associated genotyping information (reduced VCF file) to *N. fowleri* strains only, we could perform a whole-genome SNP-based phylogenetic analysis for comparing the 3 new genomes (NF_AR12, NF_PA34, and NF_NF1) against 52 strains of *N. fowleri* (close-to-complete genomes or reads only, Supplementary Table S1) available in online databases at the time of the analysis (Joseph et al., 2021). Figure 1 reveals a low level of SNP variation within the 55 *N. fowleri* isolates and we concluded that the classical typing of *N. fowleri* on the basis of their ITS sequence somehow reflects the genetic diversity of the species, which is apparently associated by geographical regions as previously established (De Jonckheere, 2011). Indeed, the newly sequenced isolates NF_AR12, NF_PA34 and the five biological replicates for NF_NF1 are located in the phylogenetic clades Genotype 2, 5, and 3, according to the numbering scheme proposed by De Jonckheere (2011), respectively, as observed using the traditional mitochondrial small subunit (mtSSU) rRNA and ITS genotyping loci (Supplementary Tables S1, S2). The SNP-based phylogenetic tree however showed no clustering between clinical and environmental isolates of *N. fowler*i strains (Figure 1; Supplementary Table S1), as recently observed by Joseph et al. (2021).

Additionally, this approach allowed us to define diagnostic SNP markers allowing to discriminate 4 genotypes of *N. fowleri*, i.e., markers whose alleles are exclusively and systematically found in all isolates of a genotype (Figure 1). More precisely, we could identify a relatively high number of specific SNPs for genotype 1 and 5 (3,744 and 298 respectively) while the number of diagnostic SNPs identified is lower for genotypes 2 and 3 (respectively 8 and 16).

### 3.2. An overview of the newly sequenced *Naegleria fowleri* and *Naegleria lovaniensis* genomes

#### 3.2.1. Genome assembly statistics and quality

Assembly statistics for the six new genomes together with the other *Naegleria* sp. whose genomes are published are presented in Table 1 and Supplementary Table S3. The six genomes have an average depth-of-coverage superior to 100X, in particular the NF_NF1 strain which benefits from the compilation of 5 replicates. The final assemblies of the new *N. fowleri* genome consist of 500, 656, and 534 scaffolds (all anchored in 37 chromosomes) with a N50 size of 125,650, 81,421 and 112,144 bp, respectively for NF_NF1, NF_AR12 and NF_PA34. Regarding the new *N. lovaniensis* genomes, we obtained for NL_F9, NL_Lova6 and NL_Lova7, 754, 1959 and 2,384 (all anchored in 37 chromosomes) with N50 values of 78,479, 26,705 and 17,708 bp, respectively. Comparison between the genome size and GC content of *N. fowleri* strains NF_ATCC 30894, NF_ATCC 30863, NF_V212, NF_986 and NF_TY and the new genomes NF_NF1, NF_AR12 and NF_PA34 show relative conservation of genome statistics. Regarding *N. lovaniensis* strain NL_ATCC 30569 and NL_76–15-250 compared to the new isolates NL_F9, NL_Lova6 and NL_Lova7, we observed that they share a similar GC content but the genome sizes for the new *N. lovaniensis* strains are smaller. As previously observed, *N. gruberi* present a slightly larger genome (40 Mb), with a 35% GC content (Table 1).

To evaluate the quality and the completeness of our assemblies, the percentage of Benchmarking Universal Single-Copy Orthologs (BUSCOs) was calculated and compared to previously sequenced *Naegleria* species (Table 1). The comparison shows globally similar numbers of complete BUSCOs within the *N. fowleri* strains, while more fragmented and missing BUSCOs could be identified in the new *N. fowleri* genomes (Table 1). Regarding the new *N. lovaniensis* genomes, the numbers of complete and fragmented BUSCOs are slightly higher than those obtained for *N. lovaniensis* NL_ATCC 30569 and NL_76–15-250. On the other hand, *N. lovaniensis* NL_F9, NL_Lova6 and NL_Lova7 present less missing BUSCOs.

#### 3.2.2. Content and organization of the genomes

To identify protein coding genes in the newly assembled *Naegleria* genomes, we performed gene prediction analyses using MAKER2 by providing evidence from transcriptomic datasets collected for each of species. Genomic features collected from the new genomes are presented in Table 1. MAKER2 gene annotation revealed that *N. fowleri* NF_NF1 and NF_AR12 have approximately the same number of genes as *N. fowleri* strain TY (*N* = 9,405), while the number of genes predicted for NF_PA34 (*N* = 11,036) is close to those predicted for the *N. fowleri* strains ATCC 30863 and strain 986 (*N* = 11,499 and 11,599, respectively). For *N. lovaniensis*, even though complete BUSCO levels are higher, the 3 new genomes present a significantly lower number of predicted genes, but the average length of the genes is higher (~3,000 bp). The repeat content is shown to be variable within *N. fowleri* strains and appears to be lower in *N. lovaniensis* new genomes (Table 1).

**Figure 2:**
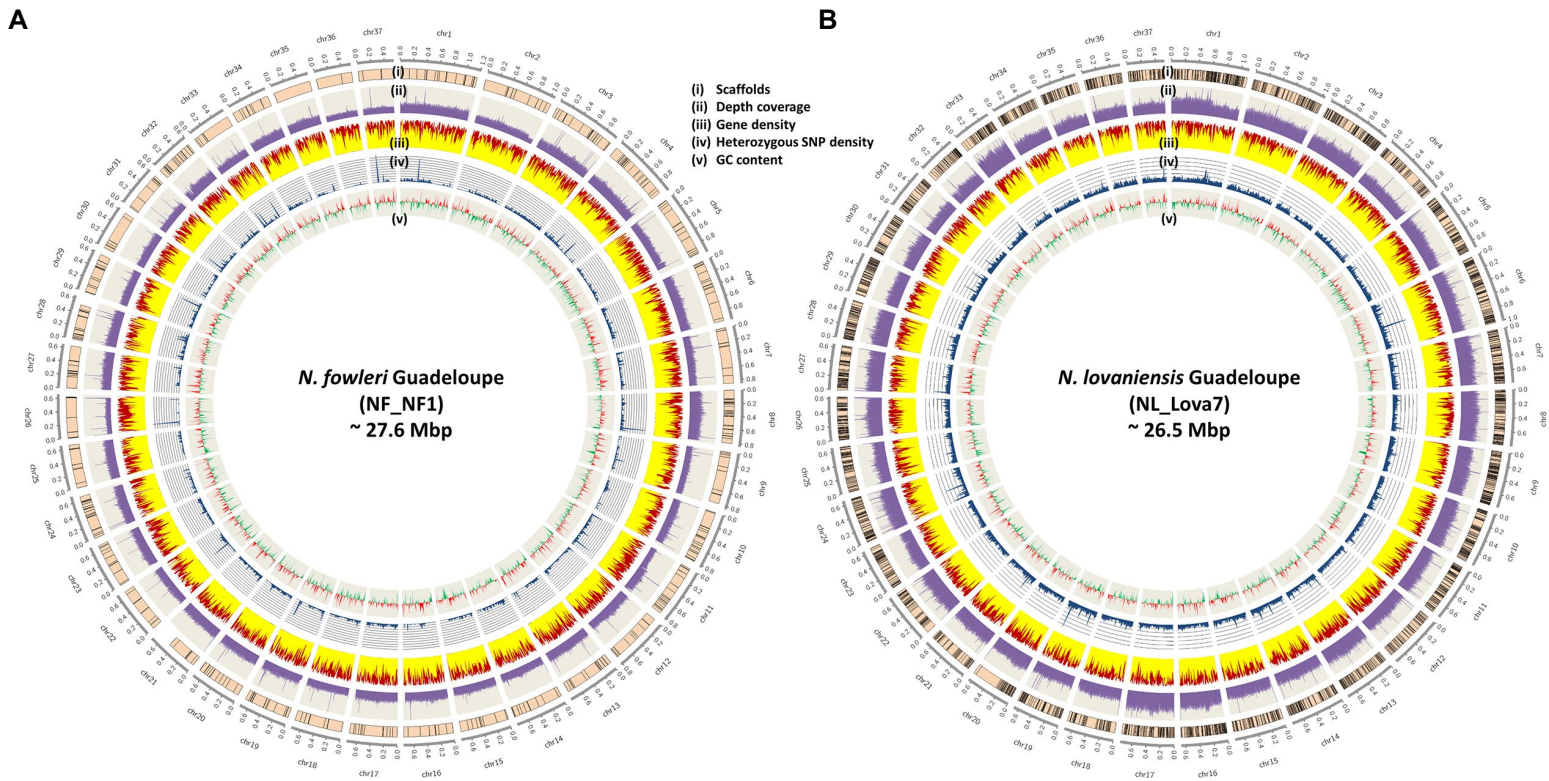
Architecture of the new genomes of *Naegleria fowleri* **(A)** and *Naegleria lovaniensis* **(B)** The outer circle represents the 37 chromosomes and their size. From outside to inside: (i) scaffolds anchoring (scaffolds were anchored and ordered using RaGOO program), (ii) sequencing depth of coverage in purple, (only one replicate (NF_NF1_1) is used for *N. fowleri,* both are plotted on a 200X scale), (iii) gene distribution (the relative part of exons and introns is colored in yellow and red respectively), (iv) heterozygous SNP density in blue and (v) GC skew (positive GCskew value in red, negative GCskew value in green). All density and distribution plots are based on 10-kb sliding windows.

The architecture of the new genomes for *N. fowleri* and *N. lovaniensis* isolated in Guadeloupe is graphically represented in Figures 2A,B, respectively. Characteristics such as scaffolds boundaries after anchoring, sequencing coverage depth, gene density (with distribution of exons and introns), heterozygous SNPs and GC content were integrated into the Circos representations. The figures show that the 2 genomes are dense in gene and exon content, with a homogenous distribution among the 37 chromosomes. In addition, we could observe variations between chromosomes in the coverage depth level, suggesting potential indirect effect of ploidy during the mapping process.

#### 3.2.3. Ploidy, aneuploidy, and loss of heterozygosity in *Naegleria*

Ploidy shifts, aneuploidy phenomena and loss of heterozygosity (LOH) have been observed in several eukaryotes (such as yeast, fungi, plants and in the parasitic amoeba *Entamoeba histolytica*; Kawano-Sugaya et al., 2020) and they have proven to be potent modulators of cell behavior, adaptation to the environment and pathogenesis (Bennett et al., 2014). The first evidence in ploidy and gene recombination in *Naegleria* was reported in 1986 using electrophoretic variation (Cariou and Pernin, 1987). In 1989, electrophoretic karyotyping showed that the number of chromosomes and their size can vary between species and even between strains of the same species (De Jonckheere, 1989).

**Figure 3:**
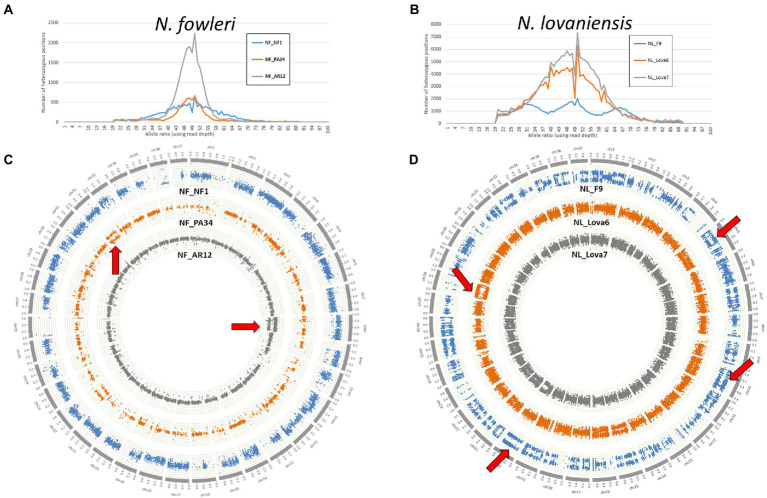
The ploidy patterns of the newly sequenced genomes from *Naegleria fowleri* and *Naegleria lovaniensis*. **(A,B)** Frequency distribution of SNP allele ratios and **(C,D)** circos scatter plots showing allelic ratios of heterozygous SNPs along chromosomes analyzed in *N. fowler*i NF_NF1, NF_PA34 and NF_AR12 strains (left panel) and *N. lovaniensis* NL_F9, NL_Lova6 and NL_Lova7 strains (right panel). Examples of suspected aneuploidy areas are highlighted by red arrows.

Herein, to assess the genomic plasticity of *Naegleria* species, we collected high probability whole genome SNPs from *N. fowleri* NF_NF1, NF_AR12 and NF_PA34 strains and *N. lovaniensis* NL_Lova6, NL_Lova7, and NL_F9 strains. We made use of the B-Allele Frequency (BAF) information, which is a normalized measure of the allelic intensity ratio of two alleles (A and B), as an indicator measure of ploidy. For the three isolates of each species, BAF measures were either reported as a distribution curve over the whole genome (number of SNPs holding BAF values) or directly plotted physically along sequences as a Circos graphical representation of the *N. fowleri* chromosomes (Figures 3A,C, respectively) or *N. lovaniensis* chromosomes (Figures 3B,D, respectively). For *N. fowleri* NF_NF1, NF_AR12 and NF_PA34 strains, we observed a peak in heterozygosity globally centered on 50% (Figure 3A), [which is consistent with the assumption that the *Naegleria* genome is diploid (Fritz-Laylin et al., 2010)], with NF_AR12 strain presenting the highest number of heterozygous positions. When analyzing the B-allele frequency (Figure 3C), we observed the *N. fowleri* strains are mainly diploid, but that trisomy phenomenon (aneuploidy with an additional chromosome) can be hypothesized visible on chr8 for NF_AR12 and chr32 for NF_PA34. This abnormality can be slightly visible also on the distributions with a slight increase to 33 and 66%. Aneuploidy is less pronounced for NF_NF1 strain, but we observed that on chr7 or chr28, the allelic ratio seems more toward 33% than toward 50%, and on the chr9 where there are slightly 2 levels of allele ratio.

For *N. lovaniensis* strains, the allele ratio fluctuates; for NL_Lova6 and NL_Lova7, we showed a major peak of allele ratios at 50%, indicating that these 2 strains are diploid with just a few triploid chromosomes observed occasionally in chr15 and chr28 in NL_Lova6, but for NL_F9, 3 peaks are observed around 33%, 50%, and 66%, suggesting that NL_F9 might be triploid on a larger number of chromosomes, reflecting several aneuploidy events (Figure 3B). Environmental conditions (such as *in vitro* culture conditions) can influence ploidy levels, as previously observed for diploid organisms such as *Saccharomyces cerevisiae* and *Candida* species (Gerstein et al., 2017). The strain NL_F9 was isolated in the 1970 and was probably more subjected to adaptative conditions, leading to an accumulation of SNP and higher variation in ploidy compared to the other strains. This increased ploidy is well observable on the Circos (Figure 3D, blue color) and it manifests notably well among others on chromosomes 5, 7, 9, 10, 11, 19, 20, 22, 25, 33.

In all strains from both species, we observed several regions of loss-of-heterozygosity (LOH), which is translated by a segmental or total loss of heterozygous SNPs. While LOH is observed in few *N. fowleri* chromosomes (chr26 for NF_NF1, chr9 for NF_PA34, chr11 for NF_AR12; Figure 3C), it is particularly widespread in the NL_F9 genome (chr4, chr13, chr23, chr28; Figure 3D).

*Naegleria* is mainly a heterozygous diploid that reproduces primarily *via* mitotic division as reviewed by De Jonckheere (2002). If the two alleles provide a differential benefit under a given stress, cells that retain the more beneficial allele after LOH may exhibit a growth advantage compared to the cells that do not undergo LOH. Extra chromosomes can arise rapidly and be lost rapidly, even within a single mitotic division. These genetic variations represent a rapid solution for adaptation to stress (Bennett et al., 2014; Peter et al., 2018).

### 3.3. *Naegleria* pangenome content

As a first step to genome functional annotation and comparative genomics, similarity searches and clustering from the 14 analyzed genomes were performed using pairwise BlastP and Orthofinder. We then analyzed two main components of the pangenome: the core genome (genes conserved across all observed genomes from a species or a genus) and accessory genome (gene(s) found at least in one strain, but not in all strains). To compare the possible advantage of using species versus genus pangenome analysis, we construct the intra-species pangenome for *N. fowleri* (*n* = 8) and *N. lovaniensis* (*n* = 5), and the *Naegleria* genus pangenome based on 14 assembled and independently annotated isolates from (i) three different species (one for *N. gruberi*, five for *N. lovaniensis* and height for *N. fowleri*), (ii) different origin (clinical and environmental, with different abilities to grow at temperatures above 37°C and with pathogenic and non-pathogenic traits in human) and (iii) from distinct geographical regions (United States, Europe, Australia, and the Caribbean).

#### 3.3.1. *Naegleria fowleri* and *Naegleria lovaniensis* species-specific pangenomes

**Figure 4:**
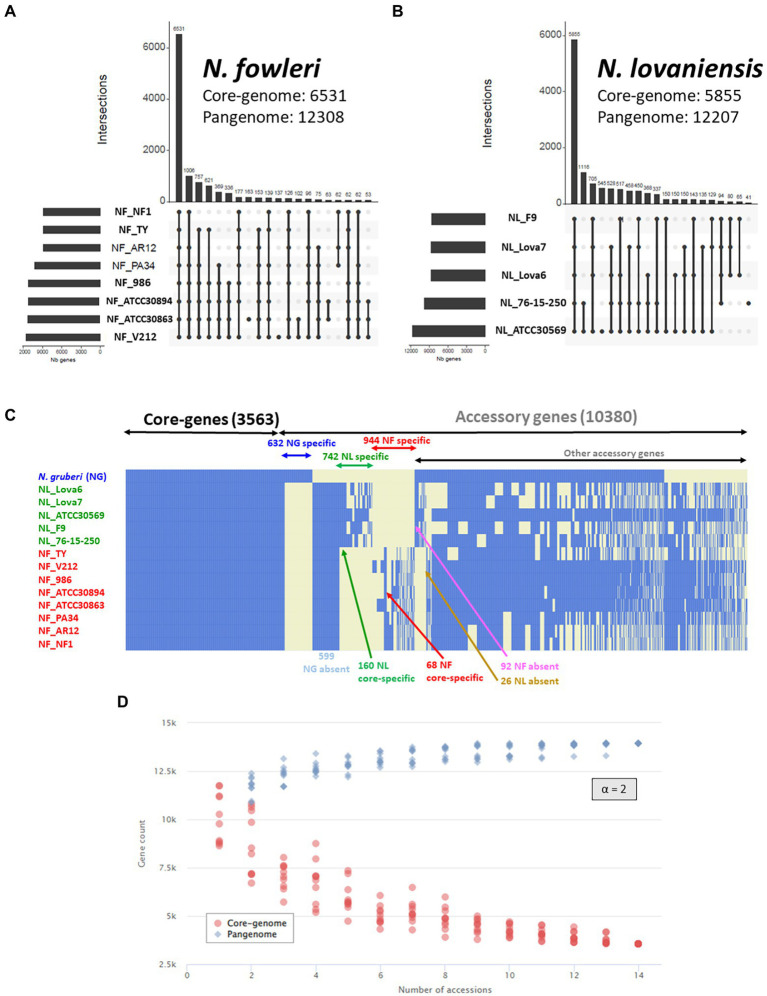
*Naegleria* pangenome analysis using OrthoFinder and visualized as UpSet and heatmap plots. UpSet plots show the intersections of the set of orthogroups from height genomes of *N. fowleri* **(A)** and five genomes of *N. lovaniensis* **(B)** species. Each vertical bar corresponds to a combination of gene sharing between strains. For each bar, black dots represent presence of the strain in the orthogroup while light gray dots denote its absence. Only the first 20 most abundant combinations are shown. The numbers of gene families (clusters/orthogroups) are indicated for each strain and strain intersection. **(C)** Gene presence/absence matrix for 14 strains of the three studied *Naegleria* species. The heatmap shows the presence (blue) or absence (light yellow) of all 13,943 orthogroups. Each row in the matrix corresponds to a strain/genome and each column represents an orthogroup. This representation has been conceived to visually separate *Naegleria* core-genes, species-specific genes and other accessory genes. **(D)** Gene accumulation curves showing how the *Naegleria* pangenome (blue) and core-genome (blue) vary as genomes are added in random order to the analysis. The power law alpha parameter shown inside the plot equals 2, which is indicative of a closed pangenome.

Genome wide statistics shows that *N. fowleri* (Figure 4A) and *N. lovaniensis* (Figure 4B) pangenomes can comprise up to 12,308 and 12,207 genes, respectively, 6,531 and 5,855 being core genes for each species. The remaining genes constitute the accessory genome, being 5,777 for *N. fowleri* and 6,352 for *N. lovaniensis*. Interestingly, Figures 4A,B reveal that two *N. fowleri* and two N. lovaniensis strains have unique genes; indeed, 183 genes were detected only in *N. fowleri* strain NF_ATCC 30863, 137 in *N. fowleri* strain NF_V212, 545 in *N. lovaniensis* strain NL_ATCC 30569 and 41 in *N. lovaniensis* strain NL_76–15-250.

The maximum proportions of genes not shared between isolates of a single species reach a maximum 10.6% of the genes for NF (for 1,006 genes, absence in Ty only), and 11.7% of the genes for NL (for 1,116 genes, found in 2 strains among 5). This rather high proportion has already been shown in other comparative genomics analyzes in other protists (Majda et al., 2019).

The Core/Pangenome ratio of *N. fowleri* and *N. lovaniensis* correspond to 53% for and 48% of the pangenome, respectively, indicating a large potential for both *Naegleria* species to adapt to their environment.

#### 3.3.2. *Naegleria* genus pangenome

Figure 4C shows that the *Naegleria* pan-genome is composed of 13,943 genes, and that the different isolates share 3,563 genes. These gene subset corresponds to the *Naegleria* core genome and provide evidence for conserved biological features among the several strains from the three species *N. gruberi*, *N. lovaniensis* and *N. fowleri*, suggesting that these genes are involved in vital role for *Naegleria* survival. The other 10,380 annotated genes correspond to the accessory genome and can be related to the evolution of a trait, speciation, or niche/host adaptation. A detailed analysis of the accessory genome allows us to detect genes exclusively present or absent in certain species (Figure 4C). For instance, we found (i) 599 genes absent in *N. gruberi*, while present in isolates from *N. lovaniensis* and *N. fowleri* and (ii) 92 genes absent in *N. fowleri* while present in both non-pathogenic *N. gruberi* and *N. lovaniensis* strains. We also looked at species-specific gene lists within accessory, regrouping 944 and 742 genes for *N. fowleri* and *N. lovaniensis, respectively.* Among these, 160 and 64 genes exclusively present in all strains of the *N. lovaniensis* and *N. fowleri,* respectively; these genes were identified as core-specific genes (i.e., species-specific and belonging to the core genome of the species).

The Core/Pangenome ratio of the *Naegleria* genus correspond to 25% of the pangenome. Compared to species-specific pangenome, our results show that the *Naegleria* core genome becomes smaller when diversity increases among the organisms. This clearly indicates that *Naegleria* community is complex and can adapt to varied niches. Additionally, the modeling of the *Naegleria* pangenome expansion show that the number of core genes (Figure 4D, red dots) and the pangenome size (Figure 4D, blue dots) stabilized after the addition of the 13 genomes, presenting an alpha parameter of 2. This demonstrates that *Naegleria* pangenome is closed (or very close to completion) and its size will not likely increase with subsequent isolates.

#### 3.3.3. Functional annotation of the *Naegleria* pangenome

**Figure 5:**
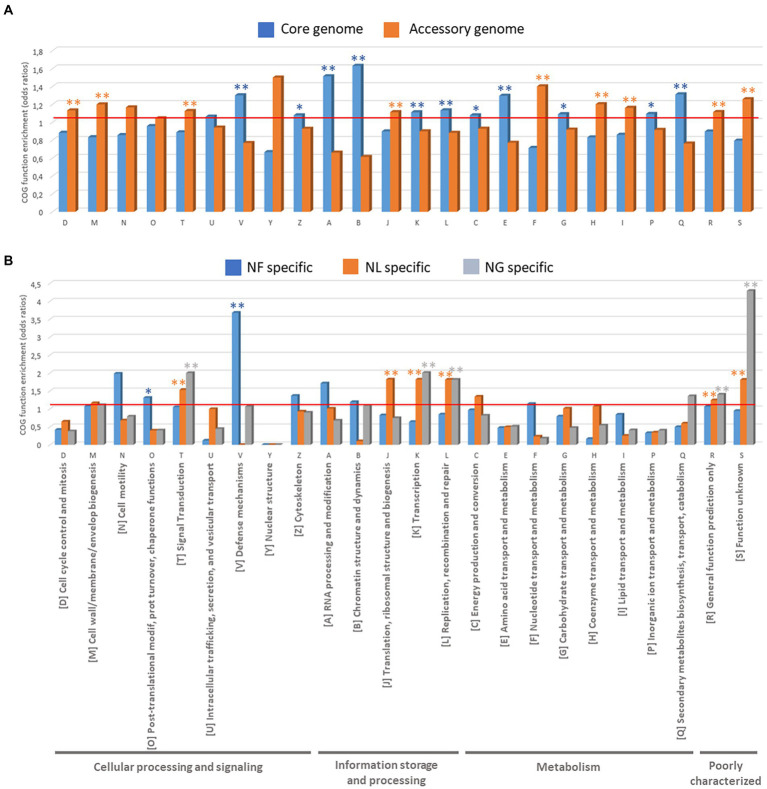
Functional analyses of *Naegleria* pangenome based on COG function enrichment (odd ratios). **(A)** COGs present in *Naegleria* core and accessory genomes and **(B)** COGs specifically detected in *N. fowleri* (NF), *N. lovaniensis* (NL) and *N. gruberi* (NG) strains.

To understand the functional roles of the genes that constitute the core and pan-genome, we used the COGs functional classification. An enrichment analysis of COG categories assigned to the genes was performed by calculating the odds-ratio values (between a particular list of genes to be compared with the rest of the genes) and associated statistics Fisher test to define enrichment if the odds-ratio is significantly superior to 1 (Figure 5).

We first compared the COG annotations assigned to the genes between the core and accessory genes in *Naegleria* sp. sequenced strains (Figure 5A; Supplementary Table S4). The *Naegleria* core genome is mainly enriched (*p* < 0.05) in protein families related to “Information storage and processing” [4 out of 5 COG categories: COG-A (RNA processing and modification), COG-B (Chromatin structure and dynamics), COG-K (Transcription) and COG-L (Replication, recombination and repair)] and “Metabolism” [5 out of 8 COG Categories: COG-C (Energy production and conversion), COG-E (Amino acid transport and metabolism), COG-G (Carbohydrate transport and metabolism) and COG-Q (Secondary metabolites biosynthesis, transport, and catabolism)] and “Cellular processing and signaling” [only 2 out of 9 COG Categories: COG-V (Defense mechanisms) and COG-Z (Cytoskeleton)]. The conservation of these shared genes among *Naegleria* species can be correlated with their biological importance in amoebae growth and survival. On the other hand, the COG functional categories of “Cellular processing and signaling” [COG-D (Cell cycle control, cell division, chromosome partitioning), COG-M (cell wall/membrane/envelope biogenesis), COG-T (Signal transduction mechanisms)], “Information storage and processing” COG-J (Translation, ribosomal structure and biogenesis) and “Metabolism” COG-F (nucleotide transport and metabolism), COG-H (Coenzyme transport and metabolism), and COG-I (Lipid transport and metabolism) are more concentrated in *Naegleria* accessory genome (Fisher *t*-test, *p* < 0.05; Figure 5A; Supplementary Table S4). Functional enrichment was not statistically significant, but *Naegleria* accessory genomes clearly showed higher proportion of genes in categories COG-N (Cell motility) and COG Y (Nuclear structure) compared to the core genome (Figure 5A). This indicates that pathways related to cell communication, ability to adapt to ecological conditions (potential niche or host-specific adaptations), genetic material processing and the metabolism of lipids and inorganic ions depend on the *Naegleria* species and/or the strain. Finally, genes within COG-S (unknown function), COG-R (general function prediction only) categories, were abundant across the pan-genome and had higher proportions in accessory genes.

To ascertain whether pathogenesis was associated with a specific functional category, we also examined the number of genes in each COG category for species-specific genes detected in pathogenic *N. fowleri* and non-pathogenic *N. lovaniensis* and *N. gruberi* (Figure 5B; Supplementary Table S4). We noticed that genes in the categories “Cellular processing and signaling” [COG-O (Post-translational modification, protein turnover, and chaperones) and COG-V (Defense mechanisms)] were remarkably enriched in *N. fowleri* strains (*p* < 0.05). Functional enrichment was not statistically significant (certainly due to the low number of genes), but still *N. fowleri* genomes clearly showed higher proportion of genes in categories COG-N (Cell motility), COG-Z (Cytoskeleton), COG-A (RNA processing and modification) and COG-B (Chromatin structure and dynamics; Figure 5B). Interestingly, our results show that non-pathogenic *N. lovaniensis* and *N. gruberi* have a similar profile of statistically relevant overexpressed proteins in COG categories T (Signal transduction mechanisms), COG-K (Transcription), COG-L (Replication, recombination and repair), and COGs R and S (poorly characterized proteins). *Naegleria lovaniensis* species-specific genes are enriched in proteins COG-J (Translation, ribosomal structure and biogenesis). Therefore, *N. fowleri* genomes shared more genes related to communication and gene control, probably allowing amoebae to respond to environmental changes more readily, while *N. lovaniensis* and *N. gruberi* genomes shared more genes involved in information storage and processing.

#### 3.3.4. Synteny between *Naegleria fowleri* and *Naegleria lovaniensis* core genomes

**Figure 6:**
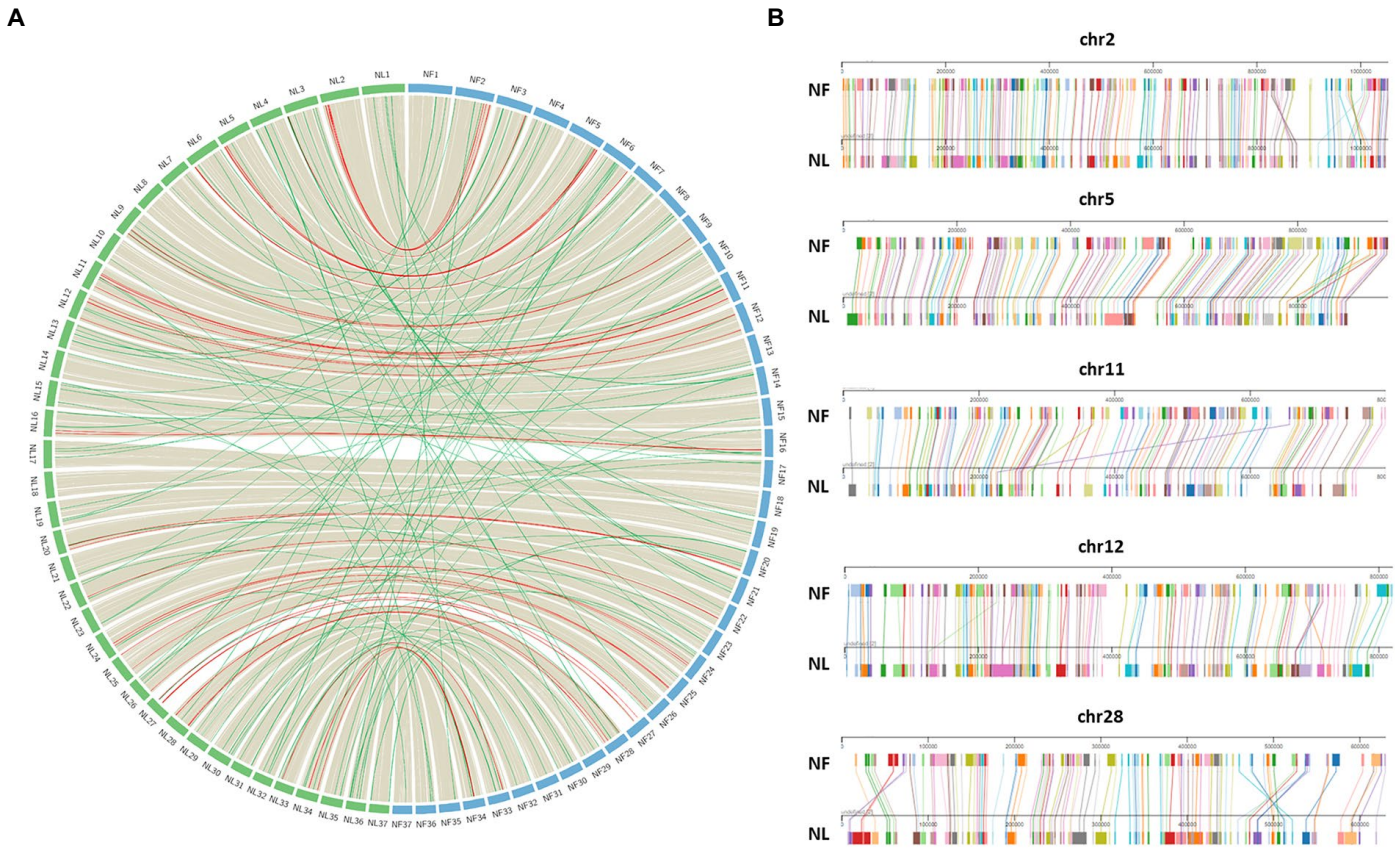
Synteny between *Naegleria fowleri* and *Naegleria lovaniensis* using core-genes. **(A)** In the circos plot, protein coding genes of *N. fowleri* (NF_NF1 strain, blue) can be connected with those of *N. lovaniensis* (NL_Lova7, green) if they belong to the core-genome. Preliminary anchoring of scaffolds using a 37 pseudo-molecules reference (NF_TY) during the assembly process, genes can be positioned onto 37 newly assembled pseudo-molecules references. Each species’ chromosome is labeled with the genus species abbreviation (NF for *N. fowleri* and NL for *N. lovaniensis*). Highly conserved syntenic blocks between the genomes are colored in gray, gene order rearrangement on the same chromosome are highlighted in red while gene order rearrangement on different chromosomes are highlighted in green. **(B)** Focus on chromosomes that show gene order rearrangement within the same chromosome using Mauve viewer. Pairwise chromosomal synteny between gene homologs of *N. fowleri* and *N. lovaniensis* in chromosomes 2, 5, 11, 12, and 28. Each colored box corresponds to a distinct core-gene that can be connected between the two reference genomes.

After establishing *Naegleria* core genome, conserved regions in terms of synteny were identified and visualized with Circos for both *N. lovaniensis* and *N. fowleri*; *N. gruberi* was not included in this analysis as it is more distant from the two other species. We observe in Figure 6A that gene synteny along the 37 chromosomes is highly conserved, with few microsyntenic breaks. Gray lines connecting core-genes with no change in gene order are in the vast majority (*n* = 4,151), while red lines (*n* = 37) highlighting regions with order changes on the same chromosome are relatively rare. For some of these cases (chr2, chr5, chr11, chr12, chr28) a zoom-in of chromosome alignment between *N. fowleri* and *N. lovaniensis* is displayed as a Mauve alignment to focus on and localize the disruption of gene order conservation (Figure 6B). Finally green lines (*n* = 88) materialize order change on different chromosomes. All together, these results showed a high conservation of gene sequences and synteny between *N. lovaniensis* and *N. fowleri*.

#### 3.3.5. *Naegleria* genes present sequence similarities with major organism groups

As a geographically widespread microorganism feeding on algae, bacteria, yeasts, and viruses in the soil and water, and with the possibility of harboring bacteria, *Naegleria* encounters a rich and diverse supply of foreign DNA, providing a large opportunity for gene exchange and acquisition (Bertelli and Greub, 2012; Sibbald et al., 2020). Herein, the functional annotation of *Naegleria* genes allowed to identify thousands of genes showing similarities with sequences from diverse kingdoms such as Animalia (including Metazoa and *Homo sapiens*), Plantae, other amoebae, Bacteria/Monera, Fungi, viruses, and other eukaryotes (Supplementary Table S4).

The social amoeba *Dictyostelium* proteins were also found to be more similar to Plants, Metazoa and Fungi (Eichinger et al., 2005). *Naegleria* genomes are known to present key features of eukaryotic origin and 2.7% of *N. gruberi* genes are homologous to bacteria (Fritz-Laylin et al., 2011; Clarke et al., 2013). This corresponds to a notably high number of laterally acquired bacterial genes compared to the parasitic *Entamoeba histolytica* or the social *Dictyostelium discoideum* (Clarke et al., 2013). In protists, fungi, plants, animals and even amoebae such as *Acanthamoeba*, *Dictyostelium* and *Naegleria*, lateral (or horizontal) gene transfer (LGT) has been considered a key process of genome evolution (Eichinger et al., 2005; Keeling and Palmer, 2008; Fritz-Laylin et al., 2010; Clarke et al., 2013; McCarthy and Fitzpatrick, 2019). Many of the LGT candidates across all of the *Naegleria* strains analyzed in this work have many predicted metabolic functions (Supplementary Table S4), suggesting that LGT in *Naegleria* might be driven by the selective pressure of new ecological niches.

Interestingly, we also noticed that *Naegleria* genes share similarities with human genes (Supplementary Table S4). Human orthologues have previously been found in the social amoeba *Dictyostelium discoideum* (Eichinger et al., 2005); and due to the strong protein homology, this amoeba is currently being used as a model to study genes related to human degenerative diseases (Haver and Scaglione, 2021). To assess if *Naegleria* could be used to investigate the functions of genes related to human disease, we performed a filtering of significant matches from Blast similarity searches between *Naegleria* and *Homo sapiens* protein sequences, using a stringent threshold E-value < 10E-20 and protein similarity extending over 65% (Table 2) with sequence coverage above 80%. Surprisingly, despite the lack of a nervous system, *Naegleria*’s genome encodes genes that cause brain and neurodegenerative diseases (Table 2), including the gene *RAB7a* (also identified in *Dictyostelium*). We also found one gene related to eye disease (which would be more related to *Acanthamoeba* species). Although orthologues of human genes implicated in disease were identified in eukaryotes (including *Dictyostelium* and yeast), *Naegleria* could provide a potentially valuable vehicle for studying their functions in a system which is experimentally tractable and intermediate in complexity between the yeasts and the higher multicellular eukaryotes *Dictyostelium*.

**Table 2:** Comparative analyses of *Naegleria* and human protein sequences.

Disease category	Annotation*	Gene name*	*Homo sapiens* accession number (Uniprot)	*BlastP alignments results*			Additional information
				Query cover (%)	Identity of coverage (%)	Probability (E-value)	
Brain disease	Cell division cycle protein 48 homolog	VCP	P55072	93	75	0	*Naegleria* core genome
	Eukaryotic translation initiation factor 2 subunit 3	EIF2S3	P41091	83	78	0	*Naegleria* core genome
	Isocitrate dehydrogenase [NADP], mitochondrial	IDH2	P48735	95%	65	0	*Naegleria* accessory genome
	14–3-3 protein epsilon	14–3-3epsilon/YWHAE	P62258	83	73	5E-136	*Naegleria* core genome
Neurodegenerative disease	Ras-related protein Rab-7A	RAB7A	P51149	96	69	6E-74	*Naegleria* core genome; also detected in *Dictyostelium discoideum*
Eye disease	Pre-mRNA-processing-splicing factor 8	PRPF8	Q6P2Q9	95	75	0	*Naegleria* core genome
Autosomal recessive metabolic disorder	Glutaryl-CoA dehydrogenase, mitochondrial	GCDH	Q92947	90	72	0	*Naegleria* accessory genome
Mitosis disorder/cancer	Serine/threonine-protein phosphatase 4 catalytic subunit	PP4C	P60510	100	78	0	*Naegleria* core genome
Intellectual disability	Thioredoxin-like protein 4A	TXN4A	P83876	100	82	1E-95	*Naegleria* accessory genome

* Based on Supplementary Table S4.

### 3.4. *Naegleria* biology and ecology

**Figure 7:**
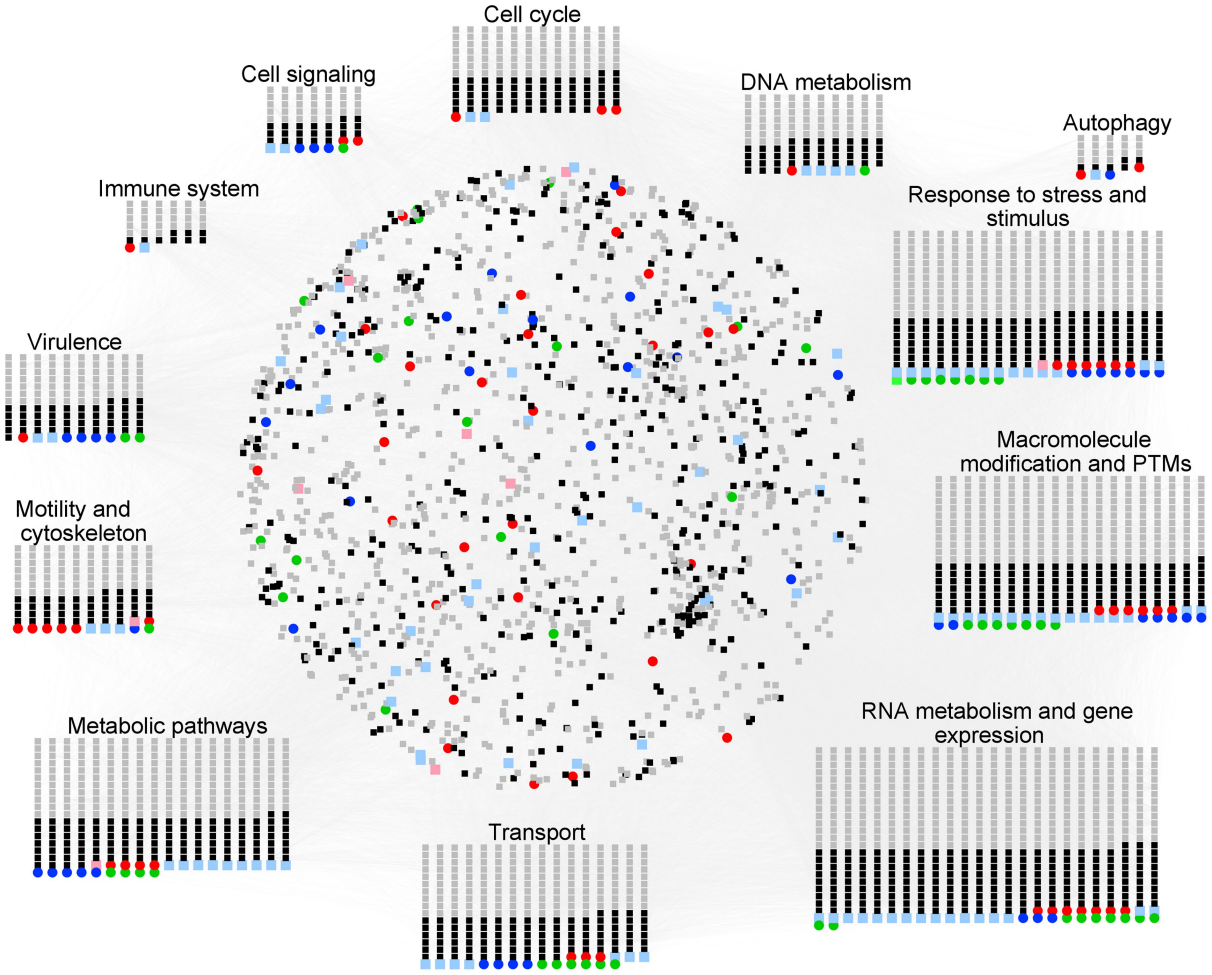
Overview of the protein–protein interaction (PPI) and functional modules identified in *Naegleria* pangenome. PPI network generated with STRING (using a high confidence score of 0.75 and visualized with Cytoscape) was built using information available in the STRING database. The nodes represent the genes, and the edges represent the protein interactions. Functional node clusters were created based on the results obtained with the STRING enrichment plugin (with a high confidence score of 0.75 and based on non-redundant terms of Gene Ontology (GO) term, Kyoto encyclopedia of genes and genomes (KEGG) pathway data and Reactome Pathways Functional Interaction (FI) Network); they highlight major biological processes in *Naegleria*. Node color code stands as red for NF, pink for genes absent in NF (but present in both NL and NG), green for NL, light green for genes absent in NL (but present in both NF and NG), blue for NG, light blue for genes absent in NG (but present in both NF and NL), black for core genes and gray for accessory (Network figures generated using https://cytoscape.org/).

**Figure 8:**
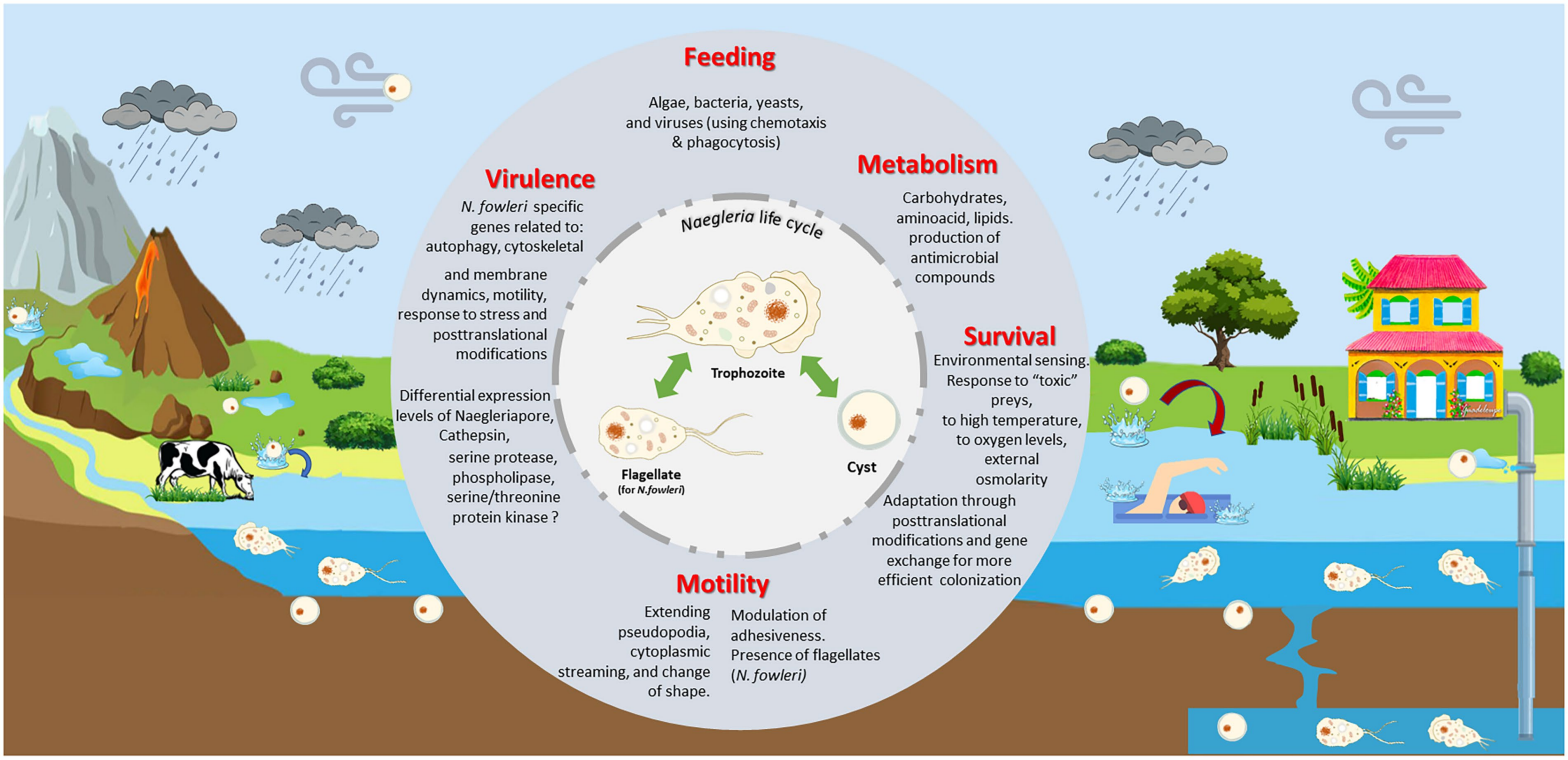
*Naegleria* biology and ecology. *Naegleria* is found worldwide in natural (such as rivers, lakes, hot springs, underground waters) and man-made (including pipes, swimming pools) aquatic environments and soil habitats. As a free-living genus, they live primarily as an amoeboid trophozoites, moving around using pseudopodia and by changing their shape. They replicate by mitosis and feed mainly on bacteria *via* phagocytosis, using chemotaxis as a possible hunting mechanism. Though they can obtain nutrients from the environments, they need to adapt their metabolism between feast and starvation conditions. Under nutrient deprivation, trophozoites can transform to resistant and dormant cysts; these can be transported by dust during dust storms. Several *Naegleria* species (namely *N. fowleri*) can exhibit a flagellate state, allowing to move around more rapidly searching for food or stressless situations. This incredible ability to swap between different forms must requires significant biochemical and genetic modifications. *Naegleria* can use other strategies to survive to stress (oxidative and osmotic stress, high temperatures, toxic preys, and predators) namely through macromolecule modification and post-translation modifications (PTMs). Genes acquired by lateral gene transfer can also provide additional help to survive in harsh situation. Few species are pathogenic but *Naegleria fowleri* trophozoites can opportunistically infect animals and humans. If water containing *N. fowleri* enters the nose, the trophozoite can enter the nasal cavity, travel to the brain and feed on the brain, beginning with the meninges. As several virulent-associated genes (such as Cathepsin and Naegleriapore pore) were found in non-pathogenic *N. gruberi* and *N. lovaniensis* and pathogenic *N. fowleri*, this suggests that high-pathogenicity of *N. fowleri* might be linked to differential expression of these genes and/or related to *N. fowleri* specific genes involved in autophagy, motility, membrane and cytoskeleton dynamics, and even PTMs.

*Naegleria* face many complex challenges in their surrounding environment. They must compete with other microorganisms for limited nutrient resources, while defending themselves against predation and toxins. The knowledge of how *Naegleria* adapt to their habitats (including during the occasional infection of the human brain) is critical for our understanding of *Naegleria* biology and pathogenesis. To move from gene content to gene context, we used Cytoscape and StringApp to construct and visualize PPI networks (Figure 7) from *Naegleria* pangenome information (Supplementary Table S4), using publicly available protein–protein-interaction information. This functional analysis allowed to infer major biological processes in *Naegleria* (Figure 7), discussed below and summarized in Figure 8.

#### 3.4.1. Life cycle and reproduction

*Naegleria* are known for their ability to form three types of cells: invasive trophozoites (amoeboid form) that divide by binary fission/mitosis, transient motile non-dividing flagellates, and latent double-walled cysts (De Jonckheere, 2002; Figure 8). The information in differential expression of genes between the different forms of *Naegleria* is very limited. Lê and co-workers found evidence that a Cystatin-B like protein (encoded by the gene mRNA1_NF0117700-p1 in NF_ATCC 30863) may play a critical role in *N. fowleri* encystation (Lê et al., 2021). We recovered Cystatin-B orthologs in *N. gruberi*, *N. fowleri* (7 out the 8 strains analyzed) and in only *N. lovaniensis* strain (Supplementary Table S5). But our results suggest that other genes might be involved in *Naegleria* encystment, which it is not fully surprising since it has been recently shown that the encystment process is a complex phenomenon, notably for the free-living amoeba *Acanthamoeba castellanii* (Bernard et al., 2022). For instance, A. *castellanii* encystment is induced by the down-regulation of the acetyltransferase-like gene *ACA1_384820* (Rolland et al., 2020). The *Naegleria* pangenome contains 26 N-acetyltransferase-like genes, 6 in the core and 20 in the accessory genome (3 being specific to *N. gruberi*; Supplementary Table S4) but none was found to be homologous to gene *ACA1_384820*. We also identified the presence of 17 genes involved in “starvation” status (and possibly involved in *Naegleria* encystment): 11 in the accessory genome, 5 in core genome, and one specific to *N. fowleri* (the gene *ULK2* coding for a Serine/threonine-protein kinase involved in autophagy in response to starvation; Supplementary Table S4). The gene coding for the transcriptional repressor *XBP1* [which is known to repress 15% of all yeast genes as cells switch to quiescence (Miles et al., 2013)] was also reported and we registered the presence of 26 orthogroups encoding for adenylate cyclase, which has been involved in encystation in *Dictyostelium* (Chen et al., 2010) and *A. castellanii* (Achar and Weisman, 1980). As observed in *Saccharomyces cerevisiae* (Yi and Huh, 2015) and previously suggested by Opperdoes et al. (2011), *Naegleria* might take advantage of the enzymes such as UTP:glucose-1-phosphate uridylyltransferase (which plays an important role in carbohydrate metabolism) and trehalose synthase and trehalose phosphatase to produce trehalose (a protectant against various abiotic stresses) for osmoregulation and cyst formation (Supplementary Tables S4, S7).

But other mechanisms could be involved in *Naegleria* encystment. *Dictyostelium discoideum* grows as a unicellular organism, but can switch to a multicellular development upon starvation (Eichinger et al., 2005). It has been suggested that *D. discoideum* transformation mechanism could be mediated by small non-coding RNA (ncRNA) mediated gene silencing (known as RNAi). Some genes involved in RNAi silencing was previously found in *N. gruberi* (Fritz-Laylin et al., 2010). Herein, elements of the RNAi machinery were found in all *Naegleria* species studied (*n* = 16, in both core and accessory genome, Supplementary Table S4). The identification of Piwi-interacting RNAs (piRNAs), a class of small RNAs that are 24–31 nucleotides in length, indicates that *Naegleria* can use gene silencing as a regulatory mechanism of gene expression. These results might encourage the use of RNAi to unravel novel aspects of *Naegleria* biology.

Despite the knowledge of the replicative ability of *Naegleria* trophozoites, little is known on the genes on *Naegleria* “sexuality.” Heterozygosity and genetic recombination found in *Naegleria gruberi* (Fritz-Laylin et al., 2010) and *N. lovaniensis* (Pernin et al., 1992) is typical of a sexual organism, and suggest some form of mating type. Meiotic and mitotic machinery was previously found in *N. gruberi* and *N. fowleri* genomes (Fritz-Laylin et al., 2010; Herman et al., 2021). Our results are in agreement with this observation as the three species studied can reproduce by mitosis (*n* = 63 genes) and they also have a complete meiosis toolkit gene (Supplementary Table S6). Indeed, we detected several genes involved in syngamy (HAP2), sister chromatid cohesion (cohesin complex, *SMC1, SM1A, DCC1, PDS5, RAD21/REC8*), formation of DNA double stranded breaks (*SPO11*, *MRE11*, and *RAD50*), homologous recombination (*DMC1*, *RAD51*, *HOP2*, and *MND1*), crossing over pathways (*MSH4-5*, *MLH1*, *MLH3*, *EXO1*, *MUS81* and *MMS4*), and gene conversion (mismatch correction, *MSH2*, *MSH6*, *PMS1-2*; Supplementary Table S6). We did not recover the genes *GEX1* [a nuclear membrane protein involved in karyogamy, also absent in *Entamoebae* (Hofstatter et al., 2018)] and *MER3* (a DNA helicase that unwinds double-stranded DNA, previously detected by Herman et al., 2021). Although the formation of the synaptonemal complex (SC) is one of the hallmarks of meiosis, we only detected one gene (SCP-2-like) potentially involved in this process. As previously observed (Fritz-Laylin et al., 2010; Herman et al., 2021), we also noticed that some of these genes (e.g., *RAD51*, *HAP2*, *MSH6*, *SPO11*) have several paralogs and certain meiosis-specific genes are differentially present in *Naegleria* species (*N. fowleri* 986 presenting the highest number of gene paralogs namely for *HAP2*, *MSH5*, *MLH1*, *MSH6* and *MLH4,* Supplementary Table S6). This variation in sexual mechanism has been previously reported for amoebozoans (Tekle et al., 2017, 2022) and *Symbiodiniaceae* dinoflagellates (Shah et al., 2020).

#### 3.4.2. Feeding and metabolism

*Naegleria* colonize environments that frequently vary in nutrient supply (carbon-, nitrogen-, phosphorus-, sulfur-sources, metal ions). To obtain nutrients such as methionine, purine and heme, the amoebae still needs to feed on various microorganisms including algae, bacteria, yeasts, and viruses (Fritz-Laylin et al., 2011). Unicellular organisms such as *Entamoeba histolytica* and *Dictyostelium discoideum* can use two complementary strategies to feed on microorganisms: chemotaxis (for detection and foraging) and phagocytosis (for recognition and digestion; Bailey et al., 1985; Xu et al., 2021). While microorganisms ‘detection and hunting are facilitated by chemoattractant G-protein-coupled-receptors (GPCRs), recognition and digestion require pattern-recognition receptors (PRRs), such as Toll-like receptors (TLRs) and C-type lectin receptors [as reviewed by Xu et al. (2021)]. Chemotaxis was previously observed in *N. fowleri* to bait for bacteria (Marciano-Cabral and Cline, 1987). *Naegleria fowleri* but not *N. australiensis*, *N. gruberi*, or *N. lovaniensis*, demonstrated enhanced motility when placed in proximity to mammalian cells (Cline et al., 1986). It has been also suggested that *N. fowleri* might actively use chemotaxis to reach the brain tissue (Baig, 2016). We also know that *Naegleria* uses mainly phagocytosis to digest bacteria, but the components involved in both chemotaxis and phagocytosis remain largely unknown. Herein, we detected 188 genes involved in *Naegleria* chemotaxis and phagocytic process: 71 in the *Naegleria* core genome and 117 in the accessory genome (7 being specific to *N. lovaniensis* strains, 3 to *N. gruberi* and 4 to *N. fowleri*; Supplementary Table S4). To sense microbial pathogens, TLRs proteins bind to bacterial elicitors through leucine-rich repeats (LRRs) and signal through adaptor/effector proteins which in turn, initiates the transcriptional programs that mediate specific defense responses (Chen et al., 2007). Herein, we identified 43 genes coding for TLRs with ligand-binding leucine-rich repeats (LRRs) in *Naegleria* core genome, 21 integrins (12 in accessory and 9 in core), C-type lectin (2 in the core genome) and 15 genes coding for concanavalin-type A lectin found in both core (*n* = 5) and accessory (*n* = 10), with one being specific to *N. lovaniensis* (OG0012291). Like human integrins, *D. discoideum* contain von Willebrand factor type A and a glycine-rich transmembrane domain and can interact with the actin-binding protein talin (Dunn et al., 2018). Herein, we found 15 genes coding for van Willebrand factor type A and interestingly only two were detected in the core genome. Among the 13 genes present in the accessory genome, 2 are specific to *N. lovaniensis* and 2 others to *N. fowleri* (Supplementary Table S4).

The phagocytotic process requires actin and cytoskeleton dynamics to accommodate the newly formed phagosome. *Naegleria gruberi* genome sequence revealed that the amoeba holds complete actin and microtubule cytoskeletons (Fritz-Laylin et al., 2010). Besides these actin-related genes, we also detected a considerable number of upstream signaling components required for cytoskeletal reorganization, such as Rho family GTPases and small GTPases. Indeed, we found that of the 25 Rho family GTPases and 111 small GTPases family detected in *Naegleria*, most of them were in the accessory, indicating that this feature is differential between the different strains or species (Supplementary Table S4). Contrary to what is observed in the social amoeba *Dictyostelium*, Cdc42 (with a profound effect on cell polarity) is specifically present in *N. lovaniensis*. The activities of these GTPases are regulated by members of the RhoGDI family, by components of ELMO1–DOCK180 complexes and by a large number of proteins carrying RhoGEF and RhoGAP domains. Herein, we found 1 gene coding for RhoGDI (in the core genome), 6 genes with ELMO domains (mainly in the accessory genome), 2 RhoGEF (in the accessory genome) and 14 RhoGAP (Supplementary Table S4). Phosphatidylinositol phosphates are crucial players during phagosome formation phagocytic uptake and maturation (Gillooly et al., 2001). As for *Dictyostelium* (Dunn et al., 2018)*, Naegleria* also possesses class I phosphatidylinositol-3-OH kinases (PI3K), which are at the crossroad of several critical signaling pathways (Supplementary Table S4). The diverse array of these regulators and the discovery of many additional actin-binding proteins suggest that there are many aspects of cytoskeletal regulation that have yet to be explored in *Naegleria*.

To breakdown several bacterial components or disrupt membrane integrity, *Naegleria* phagosome acquires a series of proteases, hydrolases, lysozymes, and antimicrobial peptides. Our results revealed the presence of 178 genes related to such pathways, with a strong homology to the genes detected in the social amoeba *Dictyostelium* (Supplementary Table S4) and with some of them being considered as virulence factors in *N. fowleri* (Herman et al., 2021). For instance, we found 19 genes coding for cathepsin proteases, including Cathepsin D (considered as a virulence factor in *N. fowleri*), a lysosomal protease involved in early phagosome steps, detected in the accessory genome. We also found that *Naegleria* is equipped with a large arsenal of lysozymes (up to 6 genes for *N. lovaniensis*, Supplementary Table S7), which suggest that each of them might be useful to kill and degrade different subsets of microorganisms. Other enzymes such as acid phosphatase, phospholipases (namely A, B, and D) and esterase [probable constituents of *Naegleria*’s phago-lysosomal system (Opperdoes et al., 2011)], chitinase, alpha and beta-amylases, alpha and beta-glucosidases, anhydro-N-acetylmuramic acid kinase and lysosomal alpha-mannosidase (that degrade bacterial peptidoglycans and glycoproteins), glucokinase, regulatory protein homolog and D-lactate dehydrogenase (involved in bacterial cell walls degradation) were also detected (Supplementary Table S7). Interestingly, the genes coding for beta-glucosidase (present in the core genome), acid phosphate (accessory genome) and lysozyme are more abundant in *N. lovaniensis.* On the other hand, the lysosomal enzyme alpha-galactosidase (involved in glycoproteins, glycolipids, and polysaccharides cleavage) was found only in some *N. fowleri* strains. Whether this relates to improved efficacy to digest bacteria or the ability to digest different bacteria genera remains to be elucidated.

As mentioned above, *Naegleria* undertake frequent transitions between feast and starvation feeding conditions, by adopting one of the three forms presented in Figure 8. All these transitions require adequate metabolic adaptations. *Naegleria*’s genomes sequencing allowed to reveal important insights into the versatility of *Naegleria’s* metabolic capacities, including the existence of aerobic and anaerobic modes of metabolism (Fritz-Laylin et al., 2011; Opperdoes et al., 2011). Our results showed that genes involved in carbohydrate and amino acid metabolism, cholesterol and sphingolipid biosynthesis, metabolism of co-factors (Supplementary Tables S4, S7) are distributed both in *Naegleria* core and accessory genomes. Although the presence of several enzymes involved in sugar transport, pentose phosphate pathways, glycolysis/gluconeogenesis suggest that *Naegleria* utilizes a variety of monosaccharides for its carbohydrate needs (Fritz-Laylin et al., 2010; Opperdoes et al., 2011; Supplementary Table S7), the role of glycolysis in *N. fowleri* during human infection remains unresolved (Milanes et al., 2019). In fact, Bexkens et al. (2018) revealed that *N. gruberi* trophozoites would prefer to oxidize fatty acids to generate acetyl-CoA, rather than use glucose and amino acids as growth substrates (Bexkens et al., 2018). Recently, several genes involved in metabolism of both lipids and carbohydrates were shown to be upregulated in mouse-passaged *N. fowleri,* being possibly related to the amoeba pathogenesis (Herman et al., 2021). Herein, we found several orthogroups encoding for Acyl-CoA synthetase (*n* = 9) which could possibly explain why lipids are more use that sugars (Supplementary Table S7). We also detected several orthogroups for adenylate kinase (*n* = 7) and succinate dehydrogenase (*n* = 4; present in all *N. lovaniensis* strains) which are implicated in energy homeostasis and production. It should be noted that *N. gruberi, N. lovaniensis* and *N. fowleri* strains are able to grow in the absence of bacteria (i.e., in axenic culture medium), in the laboratory (Cline et al., 1983; this work). Although the reasons for this are not yet clear, we suggest that, as an analogy to what was observed in the laboratory strains of *Dictyostelium*, a mutation in the gene encoding the Ras-regulating neurofibromin (that we found in the core and accessory genomes analyzed) would result in enlarged macropinosomes, and hereby facilitate the uptake of sufficient nutrients from liquid media to support growth (Bloomfield et al., 2015).

To compete for food supply and eventually inhibit the growth of the surrounding organisms, *Naegleria* might likely rely on the production of antimicrobial compounds. The analyses performed in this work revealed the presence of gene involved in the biosynthesis of secondary metabolites and resistance to antibiotics and/or other toxic compounds (Supplementary Table S4). The high number of genes encoding efflux pumps (such as ATP-binding cassette, ABC) and polyketide synthases for producing and exporting small molecules in the *Naegleria* genomes fully supports this view. ABC transporters are known to be prevalent in the proteomes of soil microorganisms such as *Dictyostelium* (Eichinger et al., 2005) and are thought to provide resistance to xenobiotics through their ability to translocate small-molecule substrates across membranes against a substantial concentration gradient. In *Naegleria*, we found 33 ABC transporters encoded by the genome (11 in the core genome and 22 others in the accessory, with one being specific to *N. fowleri* strains). In addition to polyketide synthases (*n* = 16, 5 in the core pangenome), the predicted *Naegleria* pangenome has O-methyl transferases, which could increase the diversity of natural products made. Like *Dictyostelium*, *Naegleria* appears to have a large secondary metabolism, which deserves further investigation.

#### 3.4.3. Motility

*Naegleria* display motility that is characteristic of human leukocytes (Fritz-Laylin et al., 2011). *Naegleria* trophozoites move slowly by extension of directional movements by means of broad, rounded anterior pseudopodia (lobopodia) while the flagellate possesses two basal bodies and flagella, providing a mechanism to locomote quickly in search of more favorable local conditions. The shape and locomotion of *Naegleria* are amazingly plastic.

Actin is also strongly associated to motility in *Naegleria* flagellate and amoeba stages (Velle and Fritz-Laylin, 2020). Herein, we found several genes coding for actin-related protein 2/3 complex subunit (*n* = 5), profilin (*n* = 4), WASP (*n* = 5), WAVE-complex (*n* = 2), formin (*n* = 6), and WASH-complex elements (*n* = 6; Supplementary Table S4), but most interestingly we noticed that Actin-1 [implicated in *N. fowleri* pathogenicity due to its role in trogocytosis *via* food cup formation (Sohn et al., 2010)] can be encoded by 4 (*N. lovaniensis*) to 64 genes for *N. gruberi* (Supplementary Tables S4, S5); Actin-1 can be encoded to an average of 15 genes in *N. fowleri*. The reasons for this remain unclear.

For amoeboid locomotion, the amoeba must modulate their adhesiveness to the substrate, the extracellular matrix and to other cells. To accomplish this, *Naegleria* genome encodes numerous proteins previously described as components of adherens junctions in animal Metazoa, such as β-catenin (*n* = 1, absent in *N. gruberi,* Supplementary Table S4), α-actinin (*n* = 6, one being specific to *N. fowleri*, Supplementary Table S4), myosin (*n* = 26, Supplementary Table S4), laminin (40 predicted proteins) and fibronectin. We also identified sets of genes enriched in functions specific to flagellar motility (such as dynein) in both *Naegleria* core and accessory genome. We identified 6 gene families capable of amoeboid locomotion (AMs) first detected in *N. gruberi* (Fritz-Laylin et al., 2010). Among those, only one is present in *Naegleria* core pangenome (AM46), another being specific to *N. fowleri* (AM6) and the others to accessory (Supplementary Table S4). We nevertheless noticed that *N. lovaniensis* and *N. fowleri* presented more orthogroups related to motility (namely laminin, which could represent a benefit for host tissue colonization) and in particular *N. fowleri* genomes are enriched with genes encoding proteins with leucine rich and dynein heavy chain domains (Supplementary Table S4). This higher number of genes encoding for modulation of cytoskeletal protein could be linked to *N. fowleri* pathogenesis, as previously suggest (Herman et al., 2021).

#### 3.4.4. Survival

To efficiently feed, replicate and survive, *Naegleria* must be able to sense its surrounding environments (soil, water, brain), which present different physicochemical conditions, especially in pH value and oxygen partial pressure and to compete with other amoebae (including those from the same genus), fungi and bacteria for limited resources in the different habitats. For this, *Naegleria* must have developed its gene repertoire to feed on (potentially pathogenic) microorganisms and to defend themselves against predation, toxins and environmental oxidative stress (Supplementary Table S4).

##### 3.4.4.1. Environmental sensing

*Naegleria gruberi‘s* genome encodes an extensive array of intracellular signaling machinery that presumably coordinates the environmental sensing (Fritz-Laylin et al., 2010). From Supplementary Table S4, this repertoire includes G-protein-coupled receptor signaling (*n* = 37) and histidine kinases (*n* = 17), as well as 265 predicted protein kinases, 32 protein phosphatases, and 182 monomeric Ras-like GTPases (Supplementary Table S3). Many organisms sense their environment *via* membrane-bound adenylate/guanylate cyclases; *Naegleria* contains at least 81 cyclases, 6 being specific to *N. fowleri* and 4 to *N. lovaniensis*. We also detected 4 response receiver domain proteins, whereas other protists such as *T. brucei*, *Giardia*, and *Entamoeba* have none (Fritz-Laylin et al., 2010). We also found 17 sensor protein (all homologous to bacteria) genes with PAS domain and histidine kinase domaine genes. Iron–sulfur (Fe–S) clusters have long been recognized as essential and versatile cofactors of proteins involved in sensing of ambient conditions, being essential for viability; however, these clusters can be degraded in the presence of copper. *Naegleria fowleri* has shown to be able to respond to limited iron availability (Arbon et al., 2020) and excess of copper (Grechnikova et al., 2020) to overcome oxidative stress. Herein, we detected genes coding for Fe-S cluster assembly, namely a cysteine desulfurase (with 5 orthogroups, and up to 5 gene encoding for the protein in *N. fowleri* 986), the iron chaperone frataxin and a NifU-like protein. To regulate copper levels, *Naegleria* uses copper-translocating ATPase, Selenium-binding protein 1-A, Globin/Protoglobin and Hemerythrin-like proteins (Grechnikova et al., 2020), detected in both core and accessory genome. Interestingly, the Globin/Protoglobin (OG0000194) and Hemerythrin-like protein (OG0000115) can be encoded by several genes in different *Naegleria* strains, which strongly suggest the importance of copper regulation for *Naegleria* survival.

##### 3.4.4.2. Response to “toxic” preys

During their hunt for food, *Naegleria* must often cope with the toxic traits of its prey. In aquatic environments, *Naegleria* can feed on cyanobacteria or eukaryotic algae which can expose them to photosynthetic oxidative stress (Uzuka et al., 2019). Herein, we found that *Naegleria* accessory genome encodes a gene for chlorophyllide-a oxygenase (CAO), which likely play a role in the degradation/detoxification of chlorophylls derived from prey during digestion. We also found other genes that could protect *Naegleria* from phototoxicity including RCC1 (Ultraviolet-B receptor UVR8 protein, *n* = 18), SOQ1 (*n* = 11, 5 being specific to *N. lovaniensis*) and Photoactivated adenylate cyclase (*n* = 2; Supplementary Table S3) homologous to *Euglena* genes and for photoresponsive behavior (Ntefidou and Häder, 2005).

It is widely recognized that some bacteria have evolved mechanisms to escape degradation within amoebae phagosomes (Dunn et al., 2018). In the social amoeba *D. discoideum*, as in other eukaryotic phagocytes, when bacteria escape from the phagosome, an alternative pathway to phagocytosis is triggered to eliminate infection in a more stringent catabolic way: autophagy (Mesquita et al., 2017). Herein, we detected at least 108 genes related to autophagy mechanism (Supplementary Table S3). These included previously considered *N. fowleri* virulence associated genes (*n* = 25) such as Beclin and Serine/threonine-protein kinase.

Additionally, we found evidence that *Naegleria* genome can encode at least 194 genes related to inflammatory and subsequent adaptive immune responses which supports the facts that *Naegleria* use additional defense mechanism and secretion of intercellular signals to provide a rapid antimicrobial response. Among those, 5 are specific to *N. fowleri* and 12 to *N. lovaniensis*. The genomes of the amoebae *Acanthamoeba castellani* and *Dictyostelium* are also known to encode a diverse repertoire of genes with predicted orthologous functions in the innate immune systems of higher organisms (Eichinger et al., 2005; Clarke et al., 2013).

##### 3.4.4.3. Response to high temperature

The vast majority of eukaryotes cannot survive prolonged exposure to temperatures above 40°C–45°C. Eukaryotic thermophiles (such as *N. fowleri* and *N. lovaniensis*) must have evolved to include several mechanisms of stabilization of enzymes or optimization of their activity, modulation of proportion of saturated fatty acids incorporated into phospholipids [so that their membrane fluidity is kept constant for the optimal functioning of membrane-localized transporters and enzymes (Arthur and Watson, 1976; Maheshwari et al., 2000)] and modulation of heat shock proteins. Herein, we found 97 genes involved in heat stress, two of them being in the core genome of *N. fowleri* (Supplementary Table S3).

##### 3.4.4.4. Response to oxygen levels

Although *Naegleria* present oxidative and non-oxidative modes of metabolism, when *N. fowleri* reaches the brain tissue it must possess an efficient antioxidant system to survive the invasion of oxygenated tissues and survive to the aerobic stress caused by the host immune response. Herein, we found 64 gene involved in oxidative stress. The amoebae *Mastigamoeba balamuthi* and *Entamoeba histolytica* share antioxidant system characteristics and their antioxidant machinery relies on the thioredoxin-based system (thioredoxin, NADPH: flavin oxidoreductase, peroxiredoxin), Fe-superoxide dismutase and rubrerythrin, and proteins (Žárský et al., 2021). *Naegleria* also encodes genes for thioredoxin (*n* = 21), peroxiredoxin (*n* = 3), superoxide dismutase (*n* = 4) and rubrerythrin (*n* = 1). These genes can either be found in the core and accessory genomes (Supplementary Table S3). As for *M. balamuthi*, *Naegleria* also possess (in the accessory genome) a homolog of the common bacterial osmotically inducible protein C (OsmC), which may serve as a peroxidase (Žárský et al., 2021), and multiple homologs of hemerythrin (*n* = 5, including one in the core genome) and may be involved in oxygen sensing. However, contrary to *M. balamuthi* and *E. histolytica* which lack glutathione-based pathways and catalases, we found several glutathione peroxidases that are antioxidant enzymes involved in the amoeba defense against reactive oxygen species.

##### 3.4.4.5. Response to changes in osmolality

Fluctuations in external osmolality are one of the most encountered stress signals of living cells (Saran and Schaap, 2004). Eukaryotes such as yeast and *Arabidopsis* use histidine kinases to activate mitogen-activated protein (MAP) kinase pathway after osmotic up-shift; MAP kinase pathways also mediate osmotic stress responses in animals. In *D. discoideum*, osmotic up-shift reverts the histidine kinase DokA into a histidine phosphatase, which results in inactivation of the cAMP phosphodiesterase RegA. In the amoeba spore stage, osmotic up-shift activates adenylyl cyclase G, an enzyme that is structurally homologous to the *Trypanosoma* receptor adenylyl cyclases (Saran and Schaap, 2004).

Herein, we detected several genes encoding histidine kinases (as above mentioned), MAP kinases (*n* = 30), the 3′,5′-cyclic-nucleotide phosphodiesterase regA and adenylyl cyclases (*n* = 24, 3 are specific to *N. fowleri*; Supplementary Table S3). The presence of the genes might explain the ability of *Naegleria* to survive in various water environments.

##### 3.4.4.6. Post-translational modifications

Stress responses and environmental adaptation is frequently achieved in eukaryotes through posttranslational modifications (PTMs) of signaling (Leach and Brown, 2012; Prabakaran et al., 2012; Beltrao et al., 2013). Herein, we identified for the first time, a wide repertoire of genes (at least 214 genes, 64 in core and 150 in accessory) encoding for several PTMs [such as protein SUMOylation (*n* = 4), ubiquitination (*n* = 191), neddylation (*n* = 9), Farnesylation (*n* = 10), N-and O-Mannosylation (*n* = 4); Supplementary Table S3]. This diverse variety of PTMs that might allow *Naegleria* to mount effective responses to adapt to their surroundings [including human brain, for *N. fowleri*, as previously suggested by Joseph et al. (2021)] and nutrient availability.

Ubiquitination is a reversible PTM that can modulate the activity of target proteins in various ways of numerous cellular processes, including cell cycle progression, gene transcription, DNA repair, and inflammation. Ubiquitination has been shown to be required for the survival of *S. cerevisiae* and *C. albicans* under starvation conditions (Leach and Brown, 2012). Herein, we detected 5 genes specific to *N. fowleri*, 11 to *N. lovaniensis* and 14 absent from *N. gruberi* but present in both *N. fowleri* and *N. lovaniensis*. We identified 14 E3 ubiquitin ligase (9 are specific to *N. lovaniensis* and 5 to *N. fowleri*). These enzymes are involved in the transfer of ubiquitin to substrate proteins, a process that determines the fate of the modified protein. The role mediated by E3 ligases is so crucial, that their activity must be tightly controlled to ensure they solely act when necessary. The mechanisms of protein neddylation have multiple essential functions in the cell and it appears to be important for facilitating the attachment of ubiquitin E2 to the E3 ubiquitin ligase. Cullins are a key component of cullin-RING E3 ligases, which regulate the degradation, function, and subcellular trafficking of proteins. Herein, we detected 9 genes of the cullin family of proteins. Cullin proteins were also detected in *Dictyostelium* (Kim et al., 2022).

The importance of *N-and O*-glycosylation in pathogenic fungi has been largely attributed to their key roles in the construction and maintenance of a robust cell wall, an essential structure in fungi. Herein, we also found α1,6-mannosyltransferase and Dol-P-Man:protein O-mannosyltransferases involved in O-Mannosylation. We also found evidence of GPI-anchored proteins that are also involved in *C. albicans* cell wall biosynthesis and modeling. The implication of these enzymes in *Naegleria* biology deserves further investigation.

#### 3.4.5. Virulence-associated genes

According to the initial “pangenome” concept (Tettelin et al., 2005), genes which enable the bacterial microorganisms to occupy and survive in often-hostile habitats could be considered as virulence genes. Here, we observed that many genes potentially considered as *N. fowleri* virulence associated genes (Zysset-Burri et al., 2014; Herman et al., 2021), were often found in non-pathogenic *Naegleria* strains analyzed to survive in these different habitats. These included prosaposin (termed Naegleriapore A), cathepsins B, C, L, Z, and F, serine protease, phospholipase B and serine/threonine-protein kinase.

Therefore, we specifically looked for candidate genes associated to virulence based on differences between *N. fowleri* with the non-pathogenic *N. gruberi* and *N. lovaniensis*. We could identify a set of genes exclusively present (*n* = 946) in *N. fowleri* (at least in one strain), 69 being present in all *N. fowleri* strains analyzed (Supplementary Table S4). Of these, 481 (50%) are unique to *N. fowleri*, with no clearly homologous sequence in any other organisms based on NCBI BLAST. Many of the annotated genes specific to *N. fowleri* are involved in autophagy, cytoskeletal and membrane dynamics, motility, response to stress and posttranslational modifications. Secretory products (such as glycosidase) from *N. fowleri* has been shown to play an important role in mucus degradation during the invasion process (Martínez-Castillo et al., 2017). Herein, we detected several genes coding for glycoside hydrolase (17 in core genome, 21 in accessory and 3 specific to *N. fowleri*). These enzymes could be useful to *Naegleria* to evade the mucus of the olfactory mucosa, which is part of the innate immune response (Martínez-Castillo et al., 2017). We also looked at genes specifically absent in *N. fowleri*, in particular, those potentially involved in host immune response. The absence of von Willebrand factor A domain-containing protein 3B (VWA domain-containing protein 3B) could help *N. fowleri* to escape from human immune response during infection. The presence of genes strongly homologous to human genes (Table 2) could also be useful to *N. fowleri* to go undercover in the human host.

As already discussed above, motility, proteases and lysosomal machinery have been related to *N. fowleri* pathogenesis. Recent transcriptomics experiments performed in mice suggest that up-regulation of genes involved in glutamate metabolism and ammonia transport could facilitate the spreading of *N. fowleri* in the central nervous system (Herman et al., 2021). Enzymes such as kynurenine-oxoglutarate transaminase, glutamate dehydrogenase and isocitrate dehydrogenase (involved in glutamate metabolism) and ammonium transporter were all found in the accessory genome (Supplementary Table S7) allowing the amoeba to produce brain-related neurotropic factors with impact on human mechanisms of neuroregeneration (Kim et al., 2017; Herman et al., 2021).

## 4. Conclusion

Pangenomes are becoming widely used to represent, analyze and predict the genomic diversity for populations of a single species or genus. Although the concept of the “pangenome” analysis was initially proposed in prokaryotes (Tettelin et al., 2005; Golicz et al., 2020), nowadays it is being performed in eukaryotes such as unicellular eukaryotes, fungi, plants, and animals (Aherfi et al., 2018; McCarthy and Fitzpatrick, 2019; Bayer et al., 2020; Golicz et al., 2020). In fact, pangenomics has somehow transformed eukaryote genome analyses as, regardless of their quality, eukaryote reference genomes do not and cannot contain all genetic information for a species due to genetic and genomic variation between individuals within a species or a genus (McCarthy and Fitzpatrick, 2019).

Since 2010, the number of published *Naegleria* genomes is increasing, with different levels of completeness ranging from “close-to-complete,” draft, scaffolds or reads (Fritz-Laylin et al., 2011; Liechti et al., 2018, 2019; Ali et al., 2021; Herman et al., 2021; Joseph et al., 2021). A detailed comparison of these genomes and additional functional studies using RNAseq and proteomics allowed to identify differently expressed genes potentially involved in *N. fowleri* pathogenesis (Herman et al., 2021; Joseph et al., 2021; Rodriguez-Anaya et al., 2021). Still, the understanding on how *Naegleria* can adapt to different environments, how they are phenotypically different, and why *N. fowleri* is the only pathogenic species to humans in *Naegleria* genus remains unclear.

Herein, we aimed to construct the first *Naegleria* genus pangenome, to assess the genomic repertoire of the genus and hereby open new ways to address issues related to microorganisms’ adaptation, evolution, diversity and pathogenesis (Tettelin et al., 2005; Golicz et al., 2020). For this, we presented 6 new genomes (increasing the number of genomes available for *N. fowleri* but especially for *N. lovaniensis*) and compared 14 *Naegleria* strains.

From a whole-genome SNP phylogenetic point of view, *N. lovaniensis* species displays a greater degree of variability, whereas *N. fowleri* is characterized by a phylogenetic shallowness. By defining core genomes (all genes present throughout species) and accessory genomes (strain-specific genes or genes specific to individual groups of strains), we find strong evidence for pan-genomic structure within *Naegleria*. The analysis of the pangenome of the 3 species groups revealed how they are characterized by a closed pangenome, underlining that the gene repertoire encoded by these amoebae genus is nearly complete. A more expansive analysis of the pangenome covering more genomes (including more genomes from *N. gruberi* but also other species such as *N. australiensis* and *N. italica* known to be virulent in animals) would not substantially increase the number of genes identified in this work.

*Naegleria* inhabit a wide range of soil and aquatic environments worldwide, which represents an ideal situation for gene exchanges. The functional analyses of the *Naegleria* genomes support this idea as they revealed the existence of a large fraction of genes homologous to several kingdom such as plant, animal, archaea, to bacteria and virus. The biological significance of such degree of exchange and the high number of unique genes might rely on the fact that most of amoebae have explored many possible genetic/genomic combinations, to find the more efficient phenotype for the colonization of a given ecological niche. Interestingly, *Naegleria* share orthologous genes related to human diseases. Due to a high number of conserved features comparable to Animalia (including human) and *Dictyostelium*, *Naegleria* could be a valuable and attractive tool for the study of eukaryotic cell biology and evolution. Thermophilic *Naegleria* species could also be useful to study human disease in a system which is experimentally tractable.

Successful adaptation to different habitats must require a balance between exploiting surrounding nutrients resources, competition or symbiosis with other species, replication rate and mobility efficiency. *Naegleria* has been already considered as a versatile eukaryote due its gene repertoire (Fritz-Laylin et al., 2010, 2011). Our results reveal that genomic plasticity due to changes in ploidy and aneuploidy might be underlying its ability to adapt to several environments. An important question that will need to be answered is how *Naegleria* employs these genomic strategies as a general mechanism to adapt rapidly and flexibly to changing environmental assaults.

We observed that *Naegleria* core genomes are enriched for genes that facilitate many essential metabolic, regulatory and survival processes in both non-pathogenic (*N. gruberi* and *N. lovaniensis*) and pathogenic *N. fowleri* species. Accessory genomes are enriched for genes involved in processes like gene duplication and gain/loss events within strains, and are enriched for genes involved in molecule transport, motility, immune response and proteins modification using PTMs.

While searching for a pathogenic profile in *N. fowleri,* we found virulence factors in the *Naegleria* core genome suggesting that pathogenic and non-pathogenic lifestyles might be also a result of genes expressed differentially, as it has been shown for other taxa (Meysman et al., 2013; Lòpez-Fernàndez et al., 2015). The analysis of *N. fowleri* species-specific accessory genome allowed us to detect genes that could permit increased virulence. Validation of the role of these virulence factors will require experimental confirmations.The results obtained herein suggest that drawing the line between pathogens and non-pathogenic *Naegleria* strains might be difficult, as strain-level differences in niche overlapping, ecological interactions, state of the host’s immune system and environmental factors are seldom considered. Moreover, pathogenicity must be the result of a complex, multifactorial interaction, not only dependent on qualitative issues such as the presence of specific species, strains, or genes, but also on their relative abundances (Ehrlich et al., 2008) but also genomic structure.

Globally, the characterized structural and functional divergences and similarities identified here represent an important contribution toward understanding the evolution, phenotypic diversity and versatility of the poorly studied free-living amoebae of the *Naegleria* species, paving the way for further genomic and post-genomic studies.

Because of a high number of conserved features comparable to Animalia (including human), these versatile protists could be used as a non-mammalian model to study of eukaryotic cell biology features such as resistance to temperature, cell-autonomous defense mechanisms, and host-pathogen interaction. At the moment, the non-mammalian host models predominantly used belong to the genera *Acanthamoeba* and *Dictyostelium* (phylum Amoebozoa; Eichinger et al., 2005; Chen et al., 2007; Sandström et al., 2011; Dunn et al., 2018; Swart et al., 2018; Haver and Scaglione, 2021). Although they have proven to be particularly useful to study different eukaryotic mechanisms such as host-pathogen interaction, cell motility, chemotaxis, phagocytosis, and more recently autophagy and microbiome formation, one of the major drawbacks in using these amoebae is that most of them do not grow at “elevated” temperatures such as the human body temperature. As several *Naegleria* species can withstand temperatures at 37°C (and above), these free-living amoebae could be a useful alternative to study human disease in a system which is experimentally tractable.

## Data availability statement

The data presented in the study are deposited in the figshare repository https://figshare.com/articles/dataset/NFgwada_genome_fasta/21603489 and in Genbank under BioSample accession numbers SAMN31682405, SAMN31682406, SAMN31682407, SAMN31682408, SAMN31682409 and SAMN31682410. *Naegleria* assembled chromosome sequences have been deposited on NCBI and have been assigned to accession numbers from CP113542 to CP113763. *Naegleria* ITS sequences have been deposited on NCBI and are available under accession numbers: OP867015, OP867016, OP867017 (Supplementary Table 2). Home-made scripts used for bioinformatic treatment and statistical analyses are available in a github repository accessible at https://github.com/SouthGreenPlatform/PanExplorer_workflow/tree/main/Perl/Naegleria.

## Author contributions

JJ, IM, and LM: sample preparation. AD, NA, VG, MG, LM, SJ, IA, and IM: data acquisition and analysis. AD and IM: conceptualization. AD, NA, MG, LM, IM, and VG: methodology. AD, NA, VG, and IM: formal analysis. AD, NA, VG, and IM: investigation. AD, NA, and IM: data curation. IM: writing—original draft preparation. AD, NA, VG, MG, LM, JJ, SJ, IA, AT, and IM: writing—review and editing. IM: supervision. IM and AT: project administration. AT: funding acquisition. All authors contributed to the article and approved the submitted version.

## Funding

This work was a part of the MALIN project funded by the European Union on the Guadeloupe Region under the European Research and Development Funds (ERDF) 2014–2020 program [2018-FED-1084]. C. Fund, Biomics Platform, C2RT, Institut Pasteur, Paris, France, supported by France Génomique (ANR-10-INBS-09-09) and IBISA.

## Conflict of interest

The authors declare that the research was conducted in the absence of any commercial or financial relationships that could be construed as a potential conflict of interest.

## Publisher’s note

All claims expressed in this article are solely those of the authors and do not necessarily represent those of their affiliated organizations, or those of the publisher, the editors and the reviewers. Any product that may be evaluated in this article, or claim that may be made by its manufacturer, is not guaranteed or endorsed by the publisher.

## Author disclaimer

The findings and conclusions in this report are those of the authors, and do not necessarily represent the official positions of the Centers for Disease Control and Prevention.

## Footnotes

^1^ http://www.ncbi.nlm.nih.gov/

^2^ http://www.nig.ac.jp

^3^ http://www.ncbi.nlm.nih.gov/

^4^ http://calamar.univ-ag.fr/c3i/galaxy_karubionet.html

^5^ https://amoebadb.org/amoeba/app/

^6^ https://github.com/PATRIC3/mauveviewer

^7^Namely at https://www.genome.jp/kegg
