# Supplementary figures and images for: Naegleria genus pangenome reveals new structural and functional insights into the versatility of these free-living amoebae
Source: Front Microbiol. 2023 Feb 1;13:1056418. doi: 10.3389/fmicb.2022.1056418 (PMC9928731; doi:10.3389/fmicb.2022.1056418)

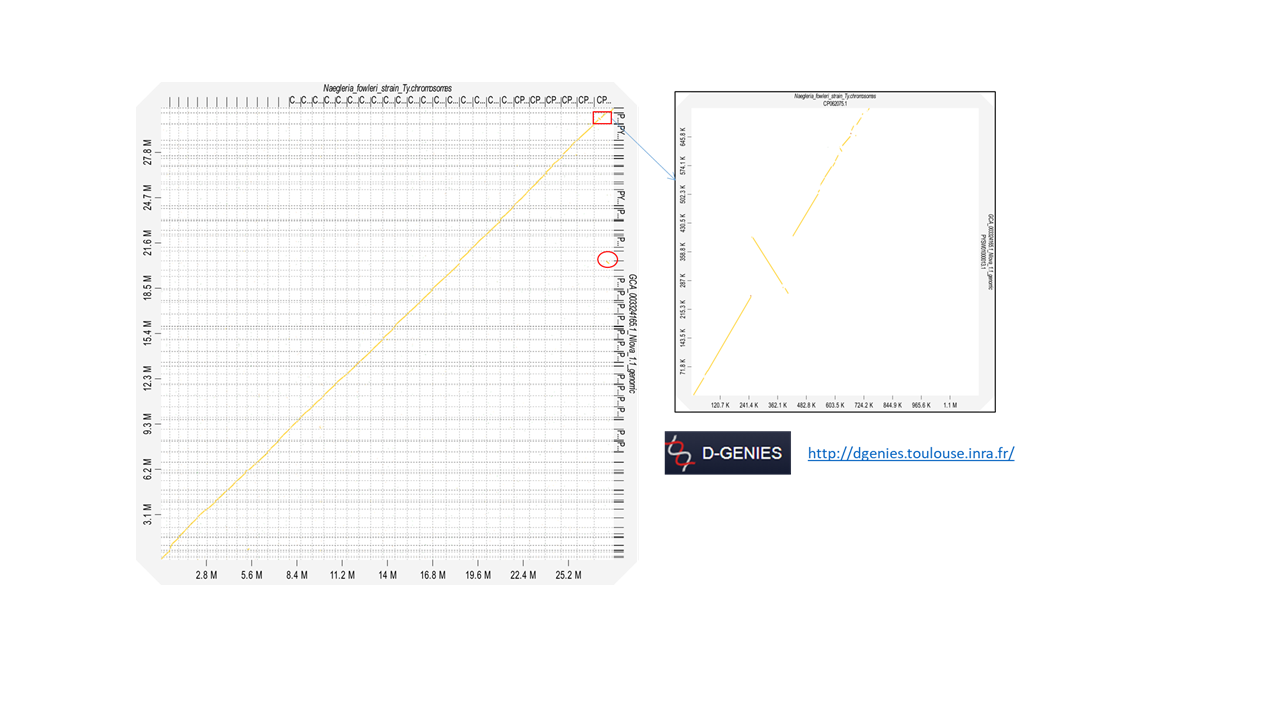

Supplement: Supplementary file 1 [file Image_1.PNG]
